# Revealing the developmental characterization of rumen microbiome and its host in newly received cattle during receiving period contributes to formulating precise nutritional strategies

**DOI:** 10.1186/s40168-023-01682-z

**Published:** 2023-11-03

**Authors:** Yanjiao Li, Kang Mao, Yitian Zang, Guwei Lu, Qinghua Qiu, Kehui Ouyang, Xianghui Zhao, Xiaozhen Song, Lanjiao Xu, Huan Liang, Mingren Qu

**Affiliations:** Jiangxi Province Key Laboratory of Animal Nutrition/Animal Nutrition and Feed Safety Innovation Team, College of Animal Science and Technology, Jiangxi Agricultural University, Nanchang, China

**Keywords:** Newly received cattle, Receiving period, Rumen-host crosstalk, Rumen metagenomics, Rumen metabolomics, Serum metabolomics, The transportation

## Abstract

**Background:**

Minimizing mortality losses due to multiple stress and obtaining maximum performance are the production goals for newly received cattle. In recent years, vaccination and metaphylaxis treatment significantly decreased the mortality rate of newly received cattle, while the growth block induced by treatment is still obvious. Assessment of blood metabolites and behavior monitoring offer potential for early identification of morbid animals. Moreover, the ruminal microorganisms’ homeostasis is a guarantee of beef steers’ growth and health. The most critical period for newly received cattle is the first-month post-transport. Therefore, analyzing rumen metagenomics, rumen metabolomics, host metabolomics, and their interaction during receiving period (1 day before transport and at days 1/4, 16, and 30 after transport) is key to revealing the mechanism of growth retardation, and then to formulating management and nutritional practices for newly received cattle.

**Results:**

The levels of serum hormones (COR and ACTH), and pro-inflammatory factors (IL-1β, TNF-α, and IL-6) were highest at day 16, and lowest at day 30 after arrival. Meanwhile, the antioxidant capacity (SOD, GSH-Px, and T-AOC) was significantly decreased at day 16 and increased at day 30 after arrival. Metagenomics analysis revealed that rumen microbes, bacteria, archaea, and eukaryota had different trends among the four different time points. At day 16 post-transport, cattle had a higher abundance of ruminal bacteria and archaea than those before transport, but the eukaryote abundance was highest at day 30 post-transport. Before transport, most bacteria were mainly involved in polysaccharides digestion. At day 4 post-transport, the most significantly enriched KEGG pathways were nucleotide metabolism (pyrimidine metabolism and purine metabolism). At day 16 post-transport, the energy metabolism (glycolysis/gluconeogenesis, pyruvate metabolism) and ruminal contents of MCP and VFAs were significantly increased, but at the same time, energy loss induced by methane yields (*Methanobrevibacter*) together with pathogenic bacteria (*Saccharopolyspora rectivirgula*) were also significantly increased. At this time, the most upregulated ruminal L-ornithine produces more catabolite polyamines, which cause oxidative stress to rumen microbes and their host; the most downregulated ruminal 2',3'-cAMP provided favorable growth conditions for pathogenic bacteria, and the downregulated ruminal vitamin B6 metabolism and serum PC/LysoPC disrupt immune function and inflammation reaction. At day 30 post-transport, the ruminal L-ornithine and its catabolites (mainly spermidine and 1,3-propanediamine) were decreased, and the serum PC/LysoPC and 2',3'-cNMPs pools were increased. This is also consistent with the changes in redox, inflammation, and immune status of the host.

**Conclusions:**

This study provides new ideas for regulating the health and performance of newly received cattle during the receiving period. The key point is to manage the newly received cattle about day 16 post-transport, specifically to inhibit the production of methane and polyamines, and the reproduction of harmful bacteria in the rumen, therefore improving the immunity and performance of newly received cattle.

Video Abstract

**Supplementary Information:**

The online version contains supplementary material available at 10.1186/s40168-023-01682-z.

## Background

In China, the north reproduction and south raise allochthonous fattening is the dominant production model of the beef industry. The health and performance of newly received cattle is still a major problem for animal welfare and economic challenges in the beef industry. Newly received cattle are exposed to various stressors and health challenges that impact their welfare and performance during the feedlot receiving period [1]. During transportation, the calves suffer dehydration, fasting, tissue damage, fume inhalation, and physical and psychological stress. After arrival, calves will experience adaptation to novel diets and environments often hinders feed intake and feed efficiency [2]. These multiple stress leads to insufficient energy supply, compromising cattle’s immune response, resulting in growth retardation or death of newly received cattle, thereby causing huge economic losses for the beef cattle industry [3, 4]. In recent years, vaccination and metaphylaxis treatment significantly decreased the mortality rate of newly received cattle, while the growth block induced by treatment is still obvious [4]. Therefore, major efforts should focus on revealing the mechanism of growth retardation in newly received cattle. Digestion and absorption are closely related to growth rate, exploring their changing regularity during the receiving period, will help develop new tailored nutritional intervention strategies to improve production.

Rumen microbes are essential for feed digestion. Rumen microbiota, including bacteria, protozoa, fungi, and archaea, are equipped with diverse enzymes able to break down complex polysaccharides of human-indigestible plant mass into volatile fatty acids (VFA), microbial proteins, and vitamins [5]. Microbial fermentation profile is accountable for up to 70% of the host’s daily energy requirements and thus is recognized as an important source of variation in cattle growth efficiency [6]. The rumen microbiota structure directly affects ruminal fermentation characteristics. Several studies showed that transport stress affects ruminal microbiota abundance and diversity of beef cattle, which further influences the fermentation profile [7–9]. Nevertheless, these studies assessed cattle rumen bacteria pre- and post-transport, whereas monitoring of total rumen microorganisms’ changes by metagenomics over receiving period is essential. Metabolomics, as the most downstream of gene expression, can more fully reflect the cell function. However, previous studies have highlighted that metabolic functions were similar even though the rumen microbiomes had different taxonomic compositions [10], suggesting that the difference in the microbiota at the composition and taxonomic levels may not be directly related to its metabolic functions that influence the host [11]. Therefore, we hypothesized that rumen metagenomics together with rumen metabolomics analysis can better elucidate the effect of stress on the rumen digestive properties of newly received cattle, and subsequently provide targeted treatment strategies to improve digestion.

Digested nutrients enter into blood circulation through the gastrointestinal epithelium to ensure growth. Several studies in beef cattle have shown that blood metabolic profile is linked to feeding efficiency [12], carcass quality [13], and live weight [14]. This means cattle growth rate was closely associated with their metabolome. Moreover, blood metabolome can quickly and objectively reflect the influence of individual variation, nutritional level, disease, and environment. While previous studies regarding the impact of transportation on blood metabolic indicators, only stress hormones, immunity, and inflammation were involved [7–9], there is no systematic analysis of changes in blood metabolites. Therefore, we further hypothesized that blood metabolomics can more comprehensively reflect the changes in the body of newly received cattle during the receiving period, thereby providing a new perspective for improving the growth performance of beef cattle. In this study, we performed rumen metagenomics, rumen metabolomics, and serum metabolomics on newly received cattle at pre- and post-transport within 1 month to address the following fundamental questions: what are the specific effects of various stresses on rumen microbes, metabolites, and host metabolism during the receiving period? What is the most significant impact? Then through the analysis of three omics, the exact strategy to improve the growth performance of the newly received cattle during the receiving period was developed. The rumen microbiome and metabolome, as well as the host metabolome, were compared 1 day before transport, days 1, 4, 6, and 30 after transport on newly received cattle, and the contributions of the above three omics layers to growth rate were calculated. The current study will provide a new window for developing nutritional management strategies to improve the production performance of introduced cattle.

## Results

### Serum parameters and rumen fermentation characteristics of newly received cattle during receiving period

**Fig. 1:**
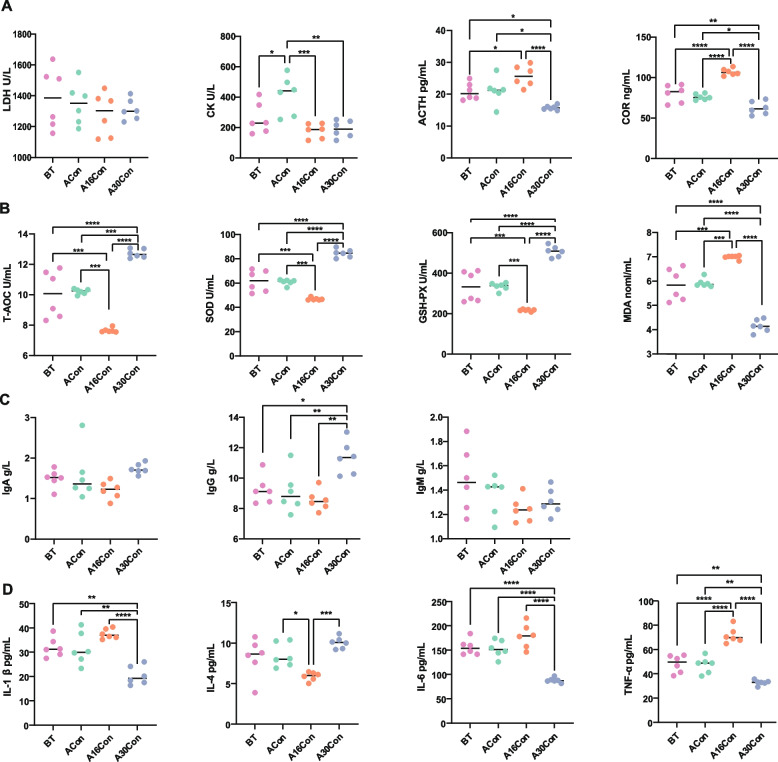
Serum parameters of newly received cattle during receiving period. **A** Stress-related enzymes and hormones indexes. **B** Antioxidant parameters. **C** Immunoglobulin factors. **D** Inflammatory factors. BT, 6 cattle of control group at 1 day before transport; ACon, 6 cattle of control group at day 1 after transport; A16Con, 6 cattle of control group at day 16 after transport; A30Con, 6 cattle of control group at day 30 after transport. ^*^P < 0.05, ^**^P < 0.01, ^***^P < 0.001, ^****^P < 0.0001. LDH = lactate dehydrogenase; CK = creatine kinase; ACTH = adreno cortico tropic hormone; COR = cortisol; T-AOC = total antioxidant capacity; SOD = superoxide dismutase; GSH-PX = glutathione peroxidase; MDA = malondialdehyde; IL-1β = interleukin-1β; IL-4 = interleukin-4; TNF-α = tumor necrosis factor-α

To explore the health status of newly received cattle during the receiving period, we analyzed serum stress-related enzymes and hormone indexes, antioxidant parameters, immunoglobulin, and inflammatory index. The creatine kinase (CK) activity was elevated at day 1 after transport and returned to normal levels at day 16 after transport. No significant change in adreno cortical tropic hormone (ACTH) and cortisol (COR) contents before transport and day 1 after transport, while their levels increased at day 16 and decreased at day 30 after transport (Fig. 1A). For antioxidant properties, the activities of total antioxidant capacity (T-AOC), superoxide dismutase (SOD) and glutathione peroxidase (GSH-PX) and malondialdehyde (MDA) content did not show significant difference before transport or day 1 after transport. However, the activities of T-AOC, SOD, and GSH-PX initially decreased at day 16 and then increased at day 30 after transport, at the same time, the MDA content showed the opposite trend (Fig. 1B). For immunoglobulins, IgA and IgM showed no significant change in levels throughout the receiving period. Also, there was no difference in IgG level from pre-transport to day 16 post-transport, while IgG level increased at day 30 post-transport (Fig. 1C). Inflammatory factors, between pre-transport and day 1 post-transport, the levels of interleukin-1β (IL-1β), IL-4, IL-6, and tumor necrosis factor-α (TNF-α) did not show significant difference. While the IL-4 level first decreased at day 16 and then increased at day 30 post-transport, at the same time, the TNF-α level showed opposite trend. Moreover, the levels of IL-1β and IL-6 were lowest at day 30 during the receiving period (Fig. 1D).

**Fig. 2:**
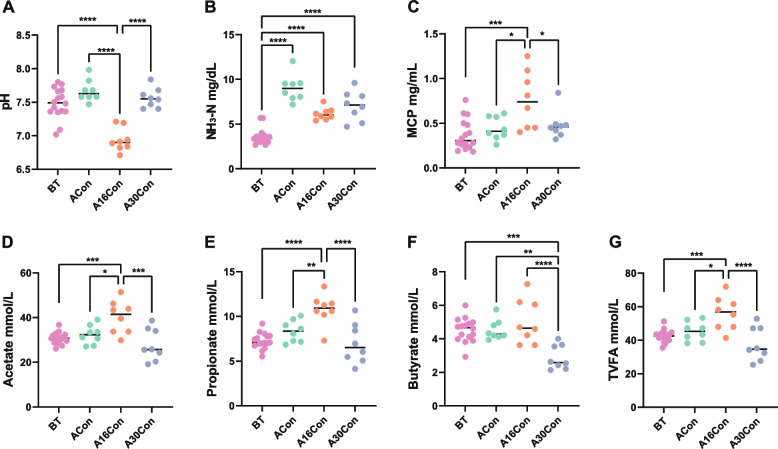
Rumen fermentation parameters of newly received cattle during receiving period. **A** Ruminal pH. **B** NH_3_-N. **C** Microbial crude protein (MCP). **D–G** Individual volatile fatty acids (VFA) and total VFA. BT, 8 cattle of control group and 8 cattle of group 4 at 1 day before transport; ACon, 8 cattle of control group at day 4 after transport; A16Con, 8 cattle of control group at day 16 after transport; A30Con, 8 cattle of control group at day 30 after transport. ^*^P < 0.05, ^**^P < 0.01, ^***^P < 0.001, ^****^P < 0.0001

To understand the variation in rumen fermentation function of newly received cattle during receiving period, ruminal pH, NH_3_-N, microbial crude protein (MCP), and VFA were measured. During the receiving period, the ruminal pH was lowest and the MCP concentration was highest at day 16 post-transport, and then ruminal pH rose and MCP concentration fell at day 30 post-transport, respectively. There were no statistically significant differences among the other time points (pre-transport, day 4 and day 30 post-transport) for ruminal pH and MCP (Fig. 2A, C). Compared with pre-transport, the ruminal NH_3_-N content was increased at days 4, 16, and 30 after transport, and its value at day 4 after transport was highest (Fig. 2B). Similar to ruminal MCP, the levels of ruminal acetate, propionate, and total VFA were highest at day 16 after transport and then decreased at day 30 after transport, also there were no statistically significant differences at the other time points (pre-transport, days 4 and 30 after transport) (Fig. 2D, E, G). The level of ruminal butyrate had a special change of lowest at day 30 after transport (Fig. 2F).

### Profiling of the rumen metagenome

Metagenome sequencing generated a total of 1,880,897,180 reads, with 78,370,716 ± 1,088,897.87 reads (mean ± standard error of the mean [SEM]) per sample (Table S2). After quality control and removing host genes, a total of 1,835,810,282 reads were retained, with 76,492,095.1 ± 1,076,103.59 per sample. After de novo assembly, a total of 20,481,942 contigs were generated (the N50 length of 764 ± 13.34 bp), with 853,414 ± 17,886.04 per sample. The rumen metagenome consisted of 97.43% bacteria (501,166,067 sequences), 0.93% eukaryotes (4,767,441 sequences), 1.16% archaea (5,957,407 sequences), 0.33% viruses (1,717,042 sequences), and 0.14% unclassified (754,032 sequences) (Fig. S1).

**Fig. 3:**
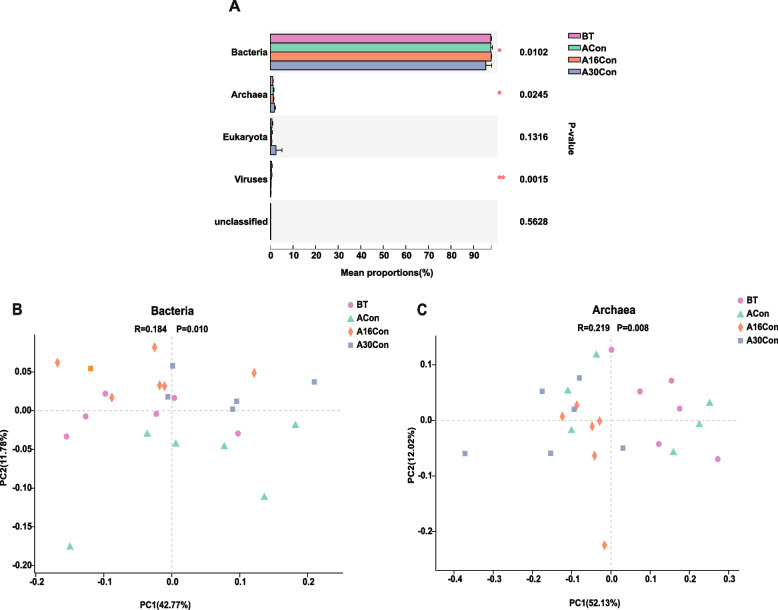
Microbial compositional profiles of newly received cattle during receiving period. **A** Comparison of microbial domains among BT, ACon, A16Con, and A30Con cattle. Significantly different domains were tested by Wilcoxon rank-sum test. ^*^P < 0.05, ^**^P < 0.01. **B** Bacterial and **C** archaeal compositional profiles of BT, ACon, A16Con, and A30Con rumen samples based on species visualized using principal coordinate analysis (PCoA). BT, 6 cattle of control group at 1 day before transport; ACon, 6 cattle of control group at day 4 after transport; A16Con, 6 cattle of control group at day 16 after transport; A30Con, 6 cattle of control group at day 30 after transport

The microbial domains were compared among the rumen microbiomes of the four groups; bacteria, archaea, and viruses were significantly different among the four groups (Fig. 3A, *P* < 0.05). The principal coordinate analysis (PCoA) showed separations among the four groups based on bacterial (Fig. 3B), archaeal (Fig. 3C), and viruses species (Fig. S2). Since sequences from viruses make up only a small portion of the rumen metagenome sequences, the statistical analysis was mainly performed on the bacterial and archaeal composition.

### Compositional profiles of the rumen microbiome and taxonomic differences among the four time points

**Fig. 4:**
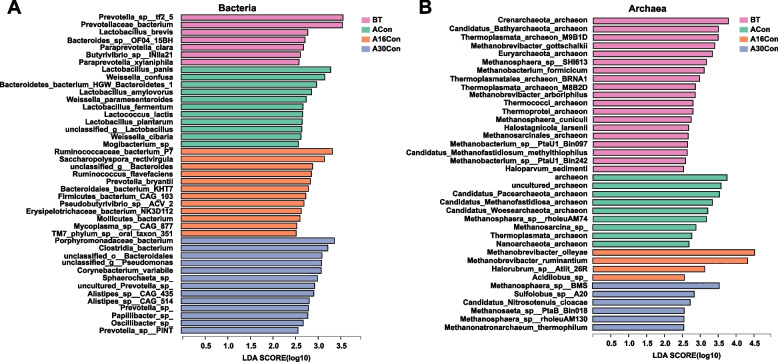
Differential rumen bacterial (**A**) and archaeal (**B**) species among BT, ACon, A16Con, and A30Con cattle according to pairwise comparison. Significant differences were tested by linear discriminant analysis effect size (LEfSe) analysis, with linear discriminant analysis (LDA) score of > 2.5 and *P* value of < 0.05. BT, 6 cattle of control group at 1 day before transport; ACon, 6 cattle of control group at day 4 after transport; A16Con, 6 cattle of control group at day 16 after transport; A30Con, 6 cattle of control group at day 30 after transport

The dominant bacterial phyla included Bacteroidetes (52.20 ± 1.67%), Firmicutes (31.00 ± 1.44%), unclassified_d_Bacteria (9.46 ± 0.52%), and Proteobacteria (1.50 ± 0.11%); the dominant bacterial genus was *Prevotella* (22.85 ± 1.65%), *Bacteroides* (4.06 ± 0.10%), unclassified*_f_Lachnospiraceae* (4.00 ± 0.22%), and unclassified*_f_Rikenellaceae* (3.78 ± 0.21%); and the dominant bacterial species included *Clostridiales_bacterium* (7.54 ± 0.47%), *Bacteroidales_bacterium* (6.58 ± 0.28%), *Rikenellaceae_bacterium* (3.78 ± 0.21%), *Prevotella_ruminicola* (3.58 ± 0.35%), and *Prevotella_sp._ne3005* (3.18 ± 0.45%). For differential abundance comparison analysis at the phylum level, the abundance of Proteobacteria was higher in the A30Con group than in the ACon and A16Con groups, and the abundance of Actinobacteria was higher in the A16Con group than in the BT group (*P* < 0.05; Fig. S3). At the genus level, the abundance of *unclassified_f__Prevotellaceae* was higher in the BT group than in the ACon group; the abundances of *unclassified_p__Firmicutes* and *unclassified_c__Clostridia* abundance were higher in the A16Con and A30Con groups than in the BT group; the *Clostridium* abundance was higher in the A16Con group than in the BT and A30Con groups; the *unclassified_f__Porphyromonadaceae* abundance was higher in the A16Con group than in the BT and ACon groups (*P* < 0.05; Fig. S3). Linear discriminant analysis effect size (LEfSe) analysis was used to detect significant differences in relative abundance of microbial species level. There were 43 differential bacterial species among four groups, 7 of which exhibited in the rumen of BT cattle, including *Prevotella_sp__tf2_5*, *Prevotellaceae_bacterium*, *Lactobacillus_brevis*, *Bacteroides_sp__OF04_15BH*, *Paraprevotella_clara*, *Butyrivibrio_sp__INlla21*, *Paraprevotella_xylaniphila* (LDA > 2.5, *P* < 0.05). Moreover, 11 species from the rumen of ACon cattle, including 5 *Lactobacillus* sp., 3 *Weissella* sp., one *Bacteroidetes* sp., one *Lactococcus* sp., and one *Mogibacterium* sp (LDA > 2.5, *P* < 0.05); 12 and 13 species were presented in the rumen of A16Con and A30Con cattle, respectively (LDA > 2.5, *P* < 0.05; Fig. 4A).

For the differential abundance comparison analysis of archaea, phyla Euryarchaeota (93.98 ± 0.85%, the most abundant archaeal phylum), and genera *Methanobrevibacter* (79.06 ± 1.76%, the most abundant archaeal genus) were significantly higher in the A16Con and A30Con groups than those in the BT and ACon groups, while the abundance of phyla Crenarchaeota (1.34 ± 0.15%) showed opposite trend (*P* < 0.01, Fig. S4). Moreover, the phyla Candidatus_Bathyarchaeota was higher in the BT group than in the A16Con and A30Con groups (*P* < 0.01, Fig. S4). At the species level, there were 38 differential species among four groups, 19, 9, 4, and 6 species showed significant enrichment in the rumen of BT, ACon, A16Con, and A30Con cattle, respectively (LDA > 2.5, *P* < 0.05; Fig. 4B). The top two differential species in BT group were *Crenarchaeota_archaeon* (1.09 ± 0.14%, the eleventh most abundant species, hereinafter only describe rank number) and *Candidatus_Bathyarchaeota_archaeon* (0.51 ± 0.08%, eighteenth); in ACon group were *archaeon* (0.91 ± 0.18%, thirteenth) and *uncultured_archaeon* (0.81 ± 0.19%, fourteenth); in A16Con group were *Methanobrevibacter_olleyae* (10.88 ± 1.03%, fourth) and *Methanobrevibacter_ruminantium* (9.34 ± 0.67%, fifth); in A30Con group were *Methanosphaera_sp._BMS* (1.02 ± 0.07%, twelfth) and *Sulfolobus_sp_A20* (over 100 most abundant species).

### Functional profiles of the rumen microbiome and differential functions among the control groups

We analyzed the functions of the rumen microbiome by the Kyoto Encyclopedia of Genes and Genomes (KEGG) profiles and genes encoding Carbohydrate-active enzymes (CAZymes). In total, 441 endogenous third-level pathways were observed through the KEGG profiles (Table S3). These pathways belonged to six level-1 categories, including Metabolism (75.87 ± 0.56%), Genetic Information Processing (8.96 ± 0.07%), Environmental Information Processing (4.40 ± 0.11%), Cellular Processes (3.99 ± 0.08%), Human Diseases (3.69 ± 0.19%), and Organismal Systems (3.10 ± 0.22%). We further picked up the level-2 key pathways, 45 categories were observed, including Global and overview maps (38.21 ± 0.29%), Carbohydrate metabolism (11.08 ± 0.08%), Amino acid metabolism (6.48 ± 0.05%), Replication and repair (4.55 ± 0.08%), Energy metabolism (3.79 ± 0.03%), and Metabolism of cofactors and vitamins (3.42 ± 0.05%) being the most abundant.

**Fig. 5:**
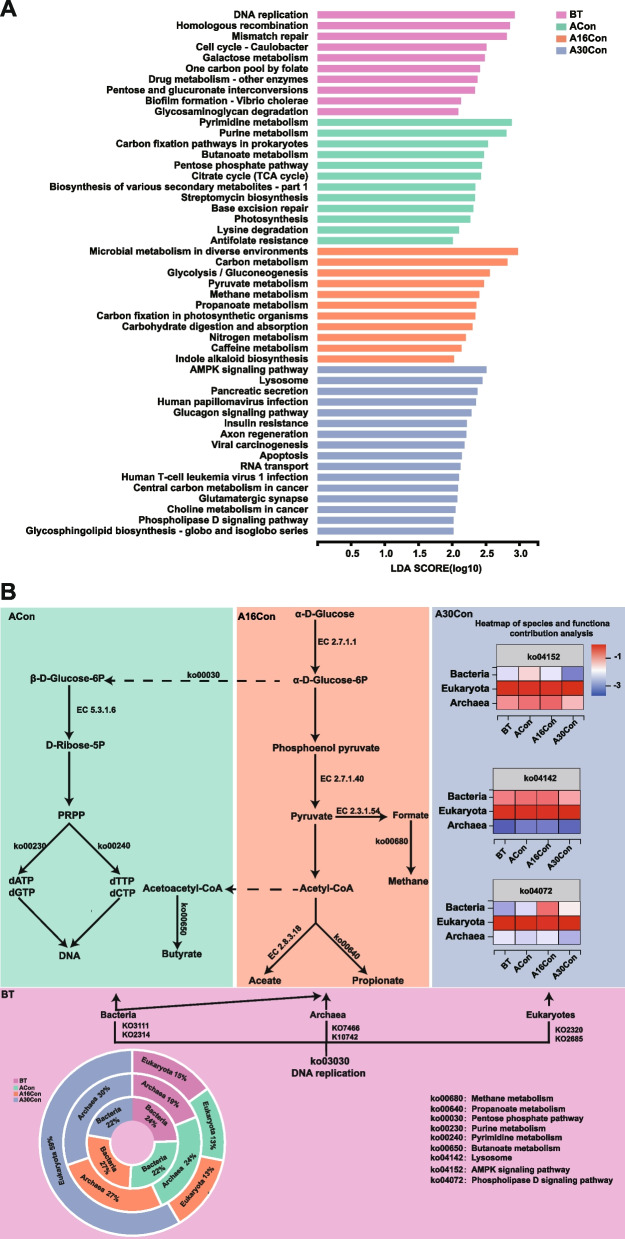
Differential KEGG functions among BT, ACon, A16Con, and A30Con cattle. **A** Comparison of rumen microbial KEGG third-level pathways among BT, ACon, A16Con, and A30Con cattle. Significant differences were tested by LEfSe analysis, with LDA score of > 2.0 and *P* value of < 0.05. **B** Consolidation of results from the rumen microbial taxa, pathways, and rumen volatile fatty acids. BT, 6 cattle of control group at 1 day before transport; ACon, 6 cattle of control group at day 4 after transport; A16Con, 6 cattle of control group at day 16 after transport; A30Con, 6 cattle of control group at day 30 after transport

When the identified KEGG pathways were compared, a total of 49 three-level pathways were significantly different among the four groups based on LEfSe analysis. Among which, 10 three-level pathways, mainly including three “Genetic Information Processing” pathways, two “Cellular Processes” pathways, and five “Metabolism” pathways were significantly enriched in the rumen of BT cattle; 12 three-level pathways, including ten “Metabolism” pathways, one “Genetic Information Processing” pathway, and one “Human Diseases” pathway were significantly enriched in the rumen of ACon cattle; 11 three-level pathways, including ten “Metabolism” pathways and one “Organismal Systems” pathway, were significantly enriched in the rumen of A16Con cattle; 16 three-level pathways, including two “Environmental Information Processing” pathways, two “Cellular Processes” pathways, four “Organismal Systems” pathways, six “Human Diseases” pathways, one “Genetic Information Processing” pathway, and one “Metabolism” pathway were significantly enriched in the rumen of A30Con cattle (LDA > 2, *P* < 0.05; Fig. 5A). Regarding “Carbohydrate metabolism” and “Energy metabolism”, two downstream pathways (ko00052: galactose metabolism, ko00040: pentose and glucuronate interconversions) were enriched in the rumen of BT cattle; five downstream pathways (ko00650: butanoate metabolism, ko00030: pentose phosphate pathway, ko00020: citrate cycle (TCA cycle), ko00720: carbon fixation pathways in prokaryotes, ko00195: photosynthesis) were enriched in the rumen of ACon cattle; six downstream pathways (ko00010: glycolysis/gluconeogenesis, ko00620: pyruvate metabolism, ko00640: propanoate metabolism, ko00680: methane metabolism, ko00710: carbon fixation in photosynthetic organisms, ko00910: nitrogen metabolism) were enriched in the rumen of A16Con cattle; there was no significant enrichment of “Carbohydrate metabolism” and “Energy metabolism” related pathways in the rumen of A30Con cattle. Moreover, notably, three “Replication and repair” downstream pathways (ko03030: DNA replication, ko03440: homologous recombination, ko03430: mismatch repair) were enriched in the rumen of BT cattle; two “Nucleotide metabolism” downstream pathways (ko00240: pyrimidine metabolism, ko00230: purine metabolism) were enriched in the rumen of ACon cattle; two “Global and overview maps” downstream pathways (ko01120: microbial metabolism in diverse environments, ko05230: carbon metabolism) were enriched in the rumen of A16Con cattle. Whereas significantly different from BT, ACon, and A16Con groups, the mostly enriched three-level pathways in the A30Con group were closely related to eukaryotic metabolism (Table S4). In sum, the significantly enriched pathways in the rumen of BT cattle mainly represent basic metabolic processes that are shared by all cells; in the rumen of ACon cattle mainly represent microbial growth and reproduction; and in the rumen of A16Con cattle mainly represent nutrition metabolism, meanwhile “Methane metabolism” was significantly enriched; in the rumen of A30Con cattle mainly related to eukaryotic metabolism (Fig. 5B).

For the Carbohydrate-active enzymes (CAZyme) profiles, a total of 534 genes encoding CAZymes were identified, which belong to 20 auxiliary activities (AAs), 73 carbohydrate-binding modules (CBMs), 16 carbohydrate esterases (CEs), 263 glycoside hydrolases (GHs), 87 glycosyltransferases (GTs), 75 polysaccharide lyases (PLs). Among them, genes encoding GH2 (7.10 ± 0.09%) were the most dominant, followed by those encoding CE1 (4.94 ± 0.09%), GT4 (4.36 ± 0.10%), CE10 (4.28 ± 0.07%), and GT41 (3.40 ± 0.06%) (Table S5). When the identified CAZymes were compared, a total of 31 family-level enzymes were significantly different among the four groups based on LEfSe analysis. Among these, 8 family-level enzymes (3 GH, 4 GT, and 1 CBM) were significantly enriched in the rumen of BT cattle; 8 family-level enzymes (3 GH, 3 GT, 1 AA, and 1 PL) were significantly enriched in the rumen of ACon cattle; 3 family-level enzymes (3 GH) were significantly enriched in the rumen of A16Con cattle; 11 family-level enzymes (11 GH and 1 CBM) were significantly enriched in the rumen of A30Con cattle (LDA > 2.5, *P* < 0.05; Fig. S5).

### LC/MS analysis of rumen and serum metabolome

**Fig. 6:**
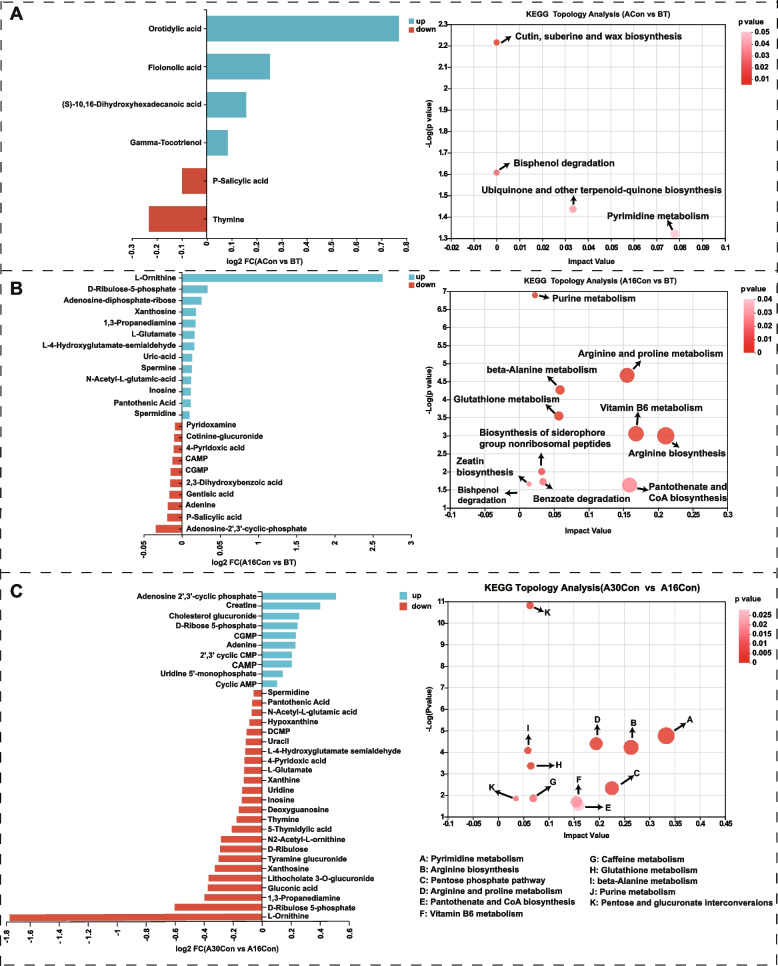
Rumen metabolome of ACon vs BT, A16Con vs BT, and A30Con vs A16Con. **A** Differential rumen metabolic enrichment pathways from ACon vs BT and ACon/BT fold change of differential metabolites involved in these pathways. **B** A16Con vs BT. **C** A30Con vs A16Con. BT, 6 cattle of control group at 1 day before transport; ACon, 6 cattle of control group at day 4 after transport; A16Con, 6 cattle of control group at day 16 after transport; A30Con, 6 cattle of control group at day 30 after transport

After rigorous quality screening and identification, we obtained 642 reliable metabolites in the ruminal fluid from the four groups. Among these, 457 metabolites were annotated in the Human Metabolome Database (HMDB) database, and the top five most abundance superclass levels include “lipids and lipid-like molecules (43.54%)”, “organic acids and derivatives (14.00%)”, “organoheterocyclic compounds (13.13%)”, “organic oxygen compounds (9.85%)”, and “nucleosides, nucleotides, and analoguesm (7.44%)” (Fig. S6). Orthogonal partial least squares discriminate analysis (OPLS-DA) was used to provide a global overview of the differences among the metabolite data (Fig. S6). All the samples in the score plots were within the 95% Hotelling T2 ellipse, and clear separation and discrimination between groups were evident. After* t* test and variable importance in projection (VIP) filtering for the relative concentrations of rumen metabolites (*P* < 0.05 and VIP > 1), 188, 225, 229, 184, 209, and 206 differential metabolites were identified from the comparisons of ACon vs BT, A16Con vs BT, A30Con vs BT, A16Con vs ACon, A30Con vs ACon, and A30Con vs A16Con, respectively (Table S6). To better understand the mechanism of metabolic pathway changes among different samples, pathway enrichment analysis of differential metabolites was performed. With 4, 11, 7, 12, 5, and 14 differential metabolic enrichment pathways from ACon vs BT, A16Con vs BT, A30Con vs BT, A16Con vs ACon, A30Con vs ACon, and A30Con vs A16on, respectively. At the same time, the ACon/BT, A16Con/BT, A30Con/BT, A16Con/ACon, A30Con/ACon, and A30Con/A16Con fold change of significantly differential rumen metabolites involved in these pathways were also displayed, respectively (Fig. 6, Fig. S7).

**Fig. 7:**
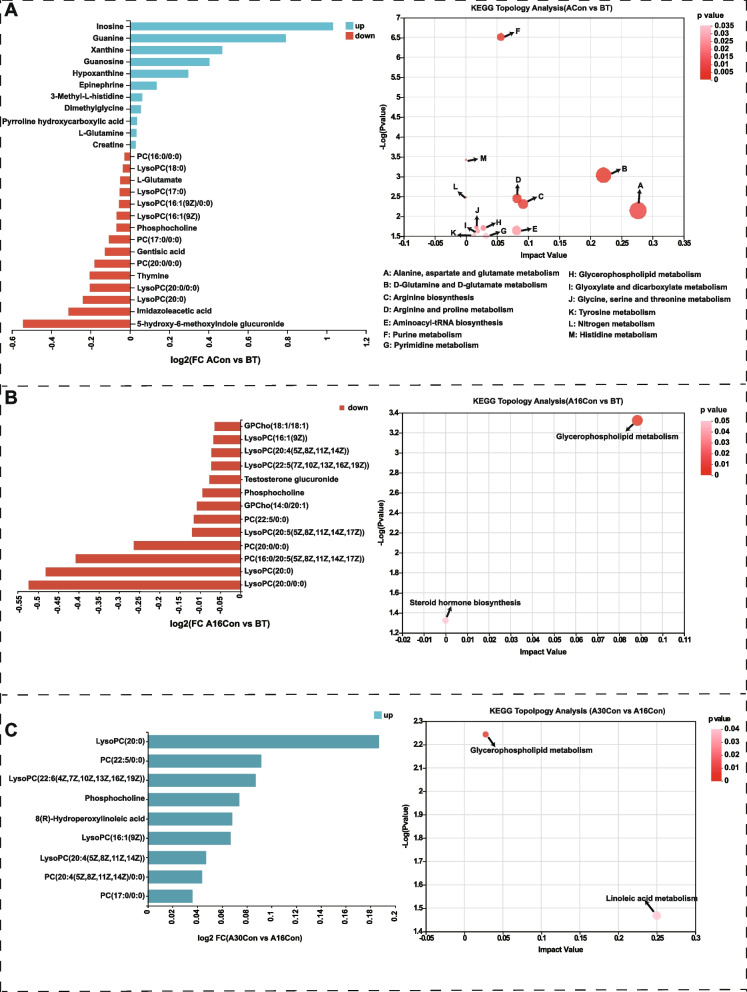
Serum metabolome of ACon vs BT, A16Con vs BT, and A30Con vs A16Con. **A** Differential serum metabolic enrichment pathways from ACon versus BT and ACon/BT fold change of differential metabolites involved in these pathways. **B** A16Con versus BT. **C** A30Con versus A16Con. BT, 6 cattle of control group at 1 day before transport; ACon, 6 cattle of control group at day 1 after transport; A16Con, 6 cattle of control group at day 16 after transport; A30Con, 6 cattle of control group at day 30 after transport

For the serum metabolome, 503 reliable metabolites were obtained from the four groups. Among these, 401 metabolites were annotated in the HMDB database, and the top five most abundance superclass levels include “lipids and lipid-like molecules (48.38%)”, “organic acids and derivatives (20.95%)”, “organoheterocyclic compounds (11.47%)”, “organic oxygen compounds (6.23%)”, and “Benzenoids (4.24%)” (Fig. S8). According to OPLS-DA score plot, it can be seen that the serum metabolites in these four groups were obviously various (Fig. S8). After* t* test and VIP filtering for the relative concentrations of serum metabolites (*P* < 0.05 and VIP > 1), there were 134, 149, 140, 178, 158, and 124 differential metabolites obtained from the comparisons of ACon vs BT, A16Con vs BT, A30Con vs BT, A16Con vs ACon, A30Con vs ACon, and A30Con vs A16Con, respectively (Table S7). There were 13, 2, 15, 10, 16, and 2 differential metabolic enrichment pathways from ACon vs BT, A16Con vs BT, A30Con vs BT, A16Con vs ACon, A30Con vs ACon, and A30Con vs A16Con, respectively. At the same time, the ACon/BT, A16Con/BT, A30Con/BT, A16Con/ACon, A30Con/ACon, and A30Con/A16Con fold change of significantly differential serum metabolites involved in these pathways were also displayed, respectively (Fig. 7, Fig. S9).

### Interactions between rumen fermentation characteristics, rumen microbiome, and rumen metabolite

**Fig. 8:**
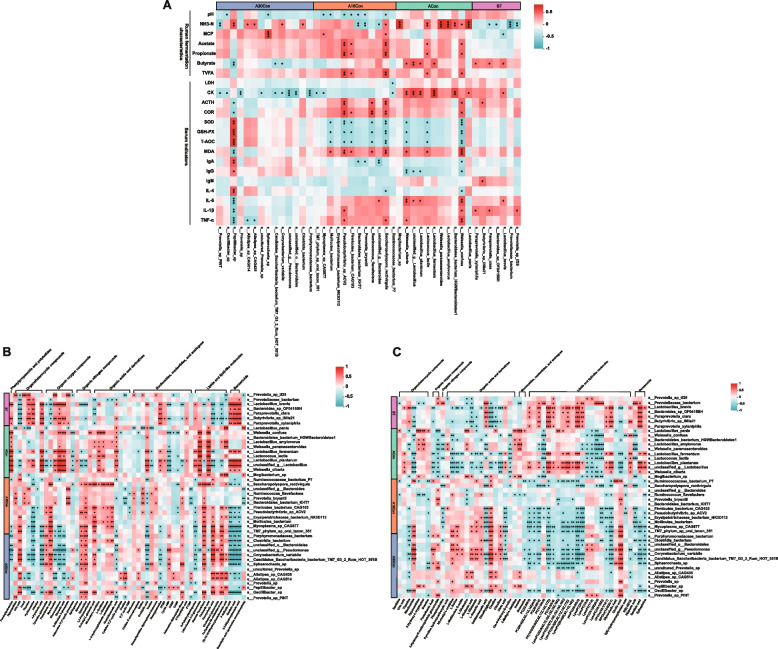
Interactions between rumen bacteria, rumen fermentation characteristic, serum indicators, rumen metabolome, and serum metabolome. **A** Spearman’s rank correlations between rumen microbiota and rumen fermentation characteristic, serum indicators. **B** Spearman’s correlations between rumen microbiota and rumen metabolites. **C** Spearman’s correlations between rumen microbiota and serum metabolites. The red mean positive correlations; the blue block mean negative correlation. “*” means a significant correlation (|*r*|> 0.5, *P* < 0.05), “**” means a strong significant correlation (|*r*|> 0.5, *P* < 0.05), “***” means the extremely significant correlation (|*r*|> 0.5, *P* < 0.05)

Spearman correlation analysis was conducted between rumen bacteria, rumen fermentation characteristic and serum indicators, with the results revealing 159 significant correlations (|R|> 0.5, *P* < 0.05), including 75 negative correlations and 84 positive correlations (Fig. 8A). Among the 159 correlations, *Riuminococcaceae bacterium P7* was negatively correlated with CK and LDH; *Alistipes sp. CAG 435* and *Alistipes sp. CAG 514* has a negative effect in TNF-α, while *Riuminococcus flavefaciens* had positive correlations with COR and ACTH, but negative correlations with serum antioxidant index (SOD, GSH-PX, T-AOC).

On the other hand, we also performed a correlation analysis between rumen bacteria and rumen metabolites, the results of heatmap show a total of 713 significant correlations (|R|> 0.5, *P* < 0.05), including 316 negative correlations and 397 positive correlations (Fig. 8B). Among the 397 positive correlations, *Saccharopolyspora_rectivirgula* was positively correlated with organic nitrogen compound (spermidine, spermine and 1, 3-propanediamine), and organic acids and derivatives (N-Acetyl-L-glutamic acid, N2-Acetyl-L-ornithine, L-Ornithine, L-Glutama).

To study the potential relationship between the host and rumen microbiome, Spearman correlation analysis was conducted between rumen bacteria and serum metabolites. As shown in Fig. 8C, there were 757 significant correlations (|R|> 0.5, *P* < 0.05), including 464 negative correlations and 293 positive correlations. Among the 464 negative correlations, rumen bacteria were negatively correlate lipids and lipid-like molecules (mainly lysophosphatidylcholine (LysoPC) and phosphatidylcholine (PC)) in A16Con, A30Con group.

## Discussion

In recent years, as the most economically significant disease of feedlot cattle, the mortality of BRD has a marked decline. However, the poor growth induced by BRD treatment is still obvious, and the detailed mechanism still needs to be undetermined. Here, we first analyzed basic blood indicators (stress markers, antioxidant parameters, immunoglobulin, and inflammatory factors) and rumen fermentation parameters to characterize the changes in health and performance in newly received cattle during receiving period. Moreover, we demonstrated the essential role of the rumen microbiome in driving rumen metabolite difference during receiving period through rumen metagenomics and metabolomics. Finally, the interaction of host metabolomics (serum metabolomics) with rumen microorganisms and their metabolites was explored to clarify the mechanisms underlying the organismal changes.

Hematological parameters can intuitively reflect the body’s condition. Numerous experiments have investigated the effects of transport stress on blood stress enzymes, stress hormones, oxidation, and immune/inflammation. However, the variations of these blood markers of newly receiving cattle during the receiving period have yet to be well reported. Therefore, in this study, we analyzed the cattle blood indicators from day 1pre-transport and then day 1, 16, and 30 post-transport. LDH and CK are enzymes that are released into the blood through leakage arising from altered membrane permeability when physical fatigue and muscle damage during handling and transport [15, 16]. Higher levels of CK and LDH enzymes in the blood induced by long distance are an indication of stress and physiological responses of animals during transportation [17]. The increased level of serum CK at day 1 post-transport (17 h after arrival) indicates that cattle have experienced significant stress during long distances and duration of transportation in this study. Another study reported an increase in cattle serum CK content just after 648 km transportation, while the CK level returned to the original values after 24 h of arrival [18]. The reason for the discrepancy between findings may be related to transport distance. The cattle in our study transported 3450 km, and the muscle damage was more excellent; therefore, the serum CK level did not return to the normal level at 17 h after transport. In response to stress, the hypothalamic–pituitary–adrenal axis and/or sympathetic nervous system were activated, which regulated the corresponding hormones secreted by the endocrine glands. Of these, ACTH and COR are often elevated in the stress reaction of cattle. Previous study showed serum ACTH and COR levels in beef cattle were increased significantly at day 1 after transport (14 h, 1000 km) and recovered to baseline levels after day 3 of arrival [7]. However, in the present study, the cattle serum ACTH and COR showed no significant difference at day one post-transport compared with pre-transport. These may be because the cattle may have adapted to long-distance transport and recovery or that adrenaline production may have been depleted during the stress period in our study. A similar situation was reported by Werner et al. [19] who found that COR level had no difference at 24 h after long-duration transportation (63 h, 1450 km). One study explored the effects of different transit times on COR levels. The results showed that higher COR levels exist in steers transported for 3 h, but not in those transported for 16 h, also supporting an adaptation to transport [20]. In the middle of the receiving period, the COR levels, whether in the second week after long-duration transportation (63 h, 1450 km) [19] or at day 15 after short-duration transportation (14 h, 1000 km) [7], were within the normal range. Inconsistently, increased levels of serum ACTH and COR at day 16 and returned to the normal levels at day 30 after transport was found in our study. There was a direct relationship between COR to feed efficiency in finishing beef cattle [21]; higher COR level means a lower feed efficiency. Thus, it is necessary to determine why ACTH and COR levels were elevated in A16Con cattle. Previous studies have found that oxidative stress affects the hypothalamic–pituitary–adrenal (HPA) axis [22], and serum ACTH and corticosterone concentrations were positively correlated with the degrees of oxidative stress [23]. Generally, to maintain the oxidative balance, cells use a suite of endogenous enzymatic and non-enzymatic antioxidants as well as antioxidants obtained exogenously to neutralize the oxidants by reduction to water [24]. The primary antioxidant enzymes within cells include SOD, GSH-PX, and catalase. An imbalance between oxidants and antioxidants can intensify oxidative stress, lead to inflammation, and compromise the health and efficiency of bovines [25]. In this study, higher MDA concentration, lower T-AOC, and decreased activities of SOD and GSH-PX existed in A16Con cattle, and the indicators recovered to the basal levels in A30Con cattle, indicating that the newly received cattle were in a state of oxidative stress at day 16 post-transport. Accordingly, it can be speculated that the higher levels of ACTH and COR in A16Con cattle may be associated with oxidative stress. Additionally, the trends in pro-inflammatory cytokines (IL-1β, IL-6, and TNF-α) were consistent with anti-inflammatory cytokines (IL-4), opposite to the trend in oxidative stress. These results suggested that oxidative stress in the mid-receiving period may be a major underlying cause of ACTH and COR concentrations, inflammatory, and immune dysfunction in newly received cattle, these may change the partitioning of energy by cattle to further impair production efficiency. What factors are contributing to the observed oxidative stress is currently unclear. Thus, we performed rumen metagenomics, rumen metabolomics, and serum metabolomics to further reveal the interaction between the rumen microbiota and host in newly received cattle at pre- and post-transport within 1 month to address the mechanisms underlying this phenomenon.

Previous work generally explored the effects of transportation on the rumen bacteria, but the lack of knowledge about the overall rumen microbiome changes during the receiving period [7–9]. In the present study, we first investigated the variation of rumen microorganisms in newly received cattle from pre-transport to 1-month post-transport by rumen metagenomics. The abundance of dominant bacteria was unchanged among BT, ACon, and A16Con groups but decreased significantly in the A30Con group, while the abundance of dominant eukaryota was increased numerically. The abundance of dominant archaea was gradually increased from the BT group to the A30Con group (Fig. 3A). DNA replication is vital for the reproduction of all organisms [26]. In order to explore the reasons behind the abundance differences observed in bacteria, archaea, and eukaryota, we performed a metabolic pathway difference-in-difference analysis on the DNA replication based on metagenomics data (Fig. S10). The results showed that helicase DnaB (K02314), primase DnaG (K02316), and single-strand DNA-binding protein (SSB, K03111), with a high relative abundance and involved in bacterial DNA replication, had highest expression levels in BT group. Therefore, it can be concluded that before transport, bacterial reproduction was more active than archaea and eukaryote. Further analysis revealed that DNA polymerase D (PolD) 1 (polB, K02323) and PolD2 (polC, K02322), primase priL (K18882), Clamp loader RfcL (K04800), and flap endonuclease-1 (Fen1, K04799), which all involved in archaeal DNA replication showed gradually increasing gene expression from BT group to A30Con group. At the same time, there was no significant difference between the A16Con group and the A30Con group. These results suggested that archaeal reproduction was relatively rapid in the middle of the receiving period and then increased slowly. Moreover, for genes related to eukaryotic DNA replication, DNA polymerase α-primase complex (Pri1, K02684), DNA polymerase δ complex (δ1, K02327; δ2, K02328), DNA polymerase ε complex (ε1, K02324; ε4, K03506), minichromosome maintenance protein (MCM) complex (helicase) (Mcm2, K02540; Mcm4, K02212; Mcm7, K02210), replication protein A (PRA) (RFA1, K07466), and Clamp loader RFC1 (K10754), demonstrated a consistent pattern of change across these genes amount in the four groups. That was, there was no significant change in the first three groups, but there was a significant increase in the fourth group, which indicated that eukaryotes thrive at the last stage of receiving period. Altogether, the reproduction of bacteria, archaea, and eukaryotes were outstanding and representative in the BT group, A16Con group, and A30Con group, respectively (Fig. 5B). The changing trend of crucial enzyme genes in DNA replication of archaea and eukaryotes were consistent with the changing trend of their abundance, but the situation in bacteria was not. For bacterial DNA replication, only the interaction of initiator protein DnaA with replication origin (OriC) unstrand the duplex unwinding element (DUE) region, the helicase (DnaB) can be recruited to the single-stranded DNA and enter the second stage. At the same time, the cellular concentration of DnaA was constant irrespective of the growth media [27]. As such, the frequency of replication initiation is primarily determined by the levels and activity of DnaA in prokaryotes [28]. In the present study, although the gene levels of helicase (DnaB) and SSB in bacterial DNA replication were gradually decreasing from the BT group to the A30Con group, the bacterial abundances remained unchanged over time and not significantly reduced until A30Con group, this may be due to the constant cellular DnaA level.

The total amount of rumen bacteria did not change among the first three groups, but the biomarkers of bacterial species shifted. The most influential different bacterial species in the BT group were *Prevotella* sp. tf2-5 and *Prevotellaceae_bacterium*. A previous study reported that the greater abundance of polysaccharide-degrading Prevotellaceae bacteria in the rumen was likely reflecting the possible need for enhanced fiber digestion [29, 30]. A recent study showed that the total tract indigestible residue (TTIR) from cattle fed a barley straw-rich diet incubated by rumen microbiota from the same cattle in vitro, the most abundant taxa of TTIR-overexpressed transcripts, including *Prevotella* (*Prevotella* sp. tf2-5, *Prevotella* sp. tc2-28) [31]. *Paraprevotella* contains only two species, *Paraprevotella clara* and *Paraprevotella xylaniphila*, which both were enriched in the rumen of BT cattle. It has been reported that a greater abundance of *Paraprevotella* in the rumen of cattle-fed cornstalk may contribute to cornstalk NDF degradation [32]. Moreover, *Lactobacillus brevis* has been reported to degrade xylo-oligosaccharides [33], *Bacteroides* sp. OF04-15BH is one of Cytophaga-Flexibacter-Bacteroides (CFB) group bacteria (NCBI: txid2292281), and CFB group bacteria are capable of degrading various biopolymers such as cellulose, chitin, and pectin [34]. In the present study, before transport, beef cattle were mainly fed on grass with complex structural polysaccharides. Therefore, the fibrolytic bacteria were the dominant bacteria. In the first 5 days after arrival, newly receiving cattle were supplemented with the probiotics and probiotic metabolites (mainly *Bacillus subtilis*, *Saccharomyces cerevisiaeits*, and their metabolites) in the drinking water (10 g/L). It has been reported that yeast-derived ingredients and *Bacillus subtilis* probiotic [35], yeast-based supplemental probiotic-prebiotic blend [36] were used as treatment and preventive measures for newly received beef cattle during the received period. Previous studies have shown that *Bacillus subtilis* and yeast culture supplementation could promote the better growth and reproduction of lactic acid bacteria in fattening cattle [37]. Consistently, in the present study, the bacteria enriched in the rumen of ACon cattle are mainly lactic acid bacteria, including five *Lactobacillus* sp. (*L. panis*, *L. amylovorus*, *L. fermentum*, *L. plantarum*, and unclassified *Lactobacillus*), three *Weissella* sp. (*W. confusa*, *W. paramesenteroides*, and *W. cibaria*), and one *Lactococcus. Lactis*. Moreover, our KEGG pathway analysis showed that pyrimidine and purine metabolism involved in nucleotide metabolism were enriched in the rumen of ACon cattle, suggesting an active microbial reproduction at this stage.

In the middle phase of the receiving period, three of the top 5 differentially abundant bacteria in the rumen of A16Con cattle were *Ruminococcaceae bacterium P7*, *Ruminococcus flavefaciens*, and *Prevotella bryantii*, which all played a pivotal role in the efficient degradation of plant cell wall polysaccharides. *Ruminococcaceae* are well-known saccharolytic bacteria to produce a range of glycosidases, enabling them to degrade hemicelluloses and cellulose [38]. Among the family *Ruminococcaceae*, the most well-studied cellulolytic species include *Ruminococcus flavefaciens*, which was originally isolated from the bovine rumen and comprises one of the three prolific cellulose degraders in the rumen [39]. *Prevotella bryantii* may ferment both structural (xylan) and nonstructural (starch) carbohydrates [40]. Correspondingly, the carbon metabolism, glycolysis/gluconeogenesis, and pyruvate metabolism were enriched in the rumen of A16Con cattle. Furthermore, rumen fermentation parameters, the contents of MCP and VFA were all the highest in this group. According to the results, there should be a sufficient energy supply for the host and further enhanced health at day 16 after arrival. Nevertheless, contrary to this expectation, the A16Con cattle had the highest levels of pro-inflammatory cytokines (IL-1β, IL-6, and TNF-α) and lowest anti-inflammatory cytokines (IL-4). In further analysis, we found that methane metabolism was also significantly enhanced in the A16Con group. The production of methane is known to result in dietary energy loss and accounts for 2–12% of the ruminant [41], thereby linking to poorer feed efficiency of the animal [42]. *Methanobrevibacter* is the most dominant methanogen in the rumen, representing up to 70% of the archaeal community [43]. Previous research showed that low feed efficiency sheep or cattle exhibited greater abundant *Methanobrevibacter olleyae* or *Methanobrevibacter ruminantium* compared to their high feed efficiency counterparts, which may partially explain a loss of energy in the low feed efficiency ruminant [44–46]. Kaplan-Shabtai et al. [47] discovered the co-occurrence of 3 distinct bacteria-archaea cohorts (methanogenic lineages) that exhibited a natural variation among dairy cows *Ruminococcus-Methanobrevibacter*, *Prevotella-Succinivibrionaceae-Methanosphaera*, and *Clostridium-Methanobacterium*. Of which, the slow-fermenting bacteria belonging to *Ruminococcaceae* and *Lachnospiraceae* form cohorts with hydrogenotrophic methanogens such as *Methanobrevibacter* lineages existed in high CH_4_-yield phenotype cows [48]. In the present study, as the fourth and fifth most abundant species within the archaea, *Methanobrevibacter olleyae* (10.88 ± 1.03%) and *M. ruminantium* (9.34 ± 0.67%) were the first two differential archaea in the rumen of A16Con cattle. Also, the relative abundance of *Ruminococcaceae bacterium P7* and *Ruminococcus flavefaciens* were highest in the rumen of A16Con cattle. Hence, it can be concluded that the *Ruminococcaceae-Methanobrevibacter* cohorts (*Ruminococcaceae bacterium P7*, *Ruminococcus flavefaciens*, *Methanobrevibacter olleyae*, and *Methanobrevibacter ruminantium*) in the rumen of A16Con cattle may produce more methane and resulting in energy waste. In addition to these bacteria and archaea, *Saccharopolyspora rectivirgula* (the second most differential bacterium) and three Mollicutes bacteria (*Erysipelotrichaceae bacterium NK3D112*, *Mollicutes bacterium*, *Mycoplasma sp. CAG 877*), reported as pathogenic bacteria, were also dominant in the rumen of A16Con cattle. *Saccharopolyspora rectivirgula* (formerly known as *Micropolyspora faeni*) exposure can cause lung infections in both bovine and bovine farmers [49]. The presence of *Saccharopolyspora rectivirgula* induces a pro-inflammatory microenvironment via the production of cytokines (IFN-γ, TNF-α, IL-1β, IL-6, IL-8, IL-10, IL-12, IL-13, IL-17A) and chemokines that recruit inflammatory cells [50]. The immunogenic *Erysipelotrichaceae* have been found to be enriched in inflammation-related disorders of the gastrointestinal tract and positively correlated with TNF-α levels [51]. Mycoplasmas are well-known pathogenic agents and most of the genus *Mycoplasma* induce disease in humans and animals [52]. Therefore, although energy metabolism (glycolysis/gluconeogenesis, pyruvate metabolism) was enhanced, the energy loss induced by higher methane yields and the pathogenic bacteria caused inflammation and oxidative stress in the A16Con cattle.

In the later receiving period in this study, the most notable feature was a significant increase in eukaryote reads in the rumen microbes of A30Con cattle, and phylum Ciliophora was dominant in the active rumen eukaryotic community. Simultaneously, the KEGG pathway analysis showed that most of the differential pathways were associated with signaling pathways in eukaryotic organisms. Rumen ciliates are known to respond rapidly and adapt to external stimuli, including the availability of nutrients [53]. Wang et al. [54] recently reported that many signaling pathways (including MAPK, mTOR, PI3K-Akt, AMPK, Wnt, calcium, and phospholipase D signaling pathway) represented in the macronuclear genome and the transcriptome of *Entodinium caudatum*, a predominant ciliate species in the rumen. The signaling pathways enable *E. caudatum* to rapidly cope with the ruminal nutritional and environmental fluctuations [55]. Besides, many transcripts were annotated to phagocytosis, phagosome, lysosome, the process and structural and functional components involved in the engulfment and digestion of microbial cells [54]. In this study, for the rumen microbial gene function in A30Con cattle, the AMPK signaling pathway and the lysosome were the top-ranking subcategories. Phospholipase D signaling pathway, which is involved in regulating membrane trafficking, cytoskeletal reorganization, receptor-mediated endocytosis, exocytosis, and cell migration [56], was also higher in A30Con cattle. They all proved to be fundamental for the growth and metabolism of protozoa. In addition, although numerically much less abundant than rumen bacteria, rumen ciliates are estimated to account for 25 to 50% of the rumen microbial biomass [57, 58]. Rumen ciliates were responsible for the breakdown of plant cell walls through their large amounts of degrading enzymes [59]. In this study, the increased abundance of phylum Ciliophora in the rumen of A30Con cattle may promote fiber digestion and feed utilization. In addition, the *Porphyromonadaceae bacterium* was the first key species responsible for the differences in the rumen of A30Con cattle. The members of *Porphyromonadaceae* can ferment various polysaccharides and mainly produce acetate and propionate [60]. *Porphyromonadaceae* have also been associated with the alleviation of colonic inflammation [61, 62]. Meanwhile, several studies confirmed that *Alistipes* abundance negatively correlated with the inflammatory cytokine levels [63, 64]. In the present study, the results of correlation analysis show that *Alistipes sp. CAG 435* and *Alistipes sp. CAG 514* have a negative effect on TNF-α (Fig. 8A). Therefore, the reduced levels of inflammation and oxidative stress in the A30Con cattle may be associated with a higher amount of rumen ciliates and anti-inflammatory bacteria.

Rumen metabolomics supported microbial functional genomic interpretations; serum metabolomics can reveal the interaction mechanism between the rumen host and microbes and their metabolites. Firstly, a comparison of the rumen metabolites of ACon vs BT showed that the orotidylic acid concentration was higher in ACon. Orotidylic acid (also known as orotidine-5'-phosphate or orotate monophosphate (OMP)) is an intermediate in the de novo pyrimidine biosynthesis pathway and converted from orotate and 5-phosphoribosyl-1-pyrophosphate (PRPP) by orotate phosphoribosyl transferase. The dehydrogenation of dihydroorotate to orotate is the fourth and rate-limiting step of the pyrimidine biosynthesis pathway catalyzed by dihydroorotate dehydrogenase ([EC:1.3.98.1]) [65]. Consistently, the gene expression of rumen microbial dihydroorotate dehydrogenase of ACon cattle was highest (Fig. S11). As opposed to elevated orotidylic acid, the content of thymine was decreased. These may be due to that thymine was converted to thymidine catalyzed by nucleoside deoxyribosyltransferase ([EC:2.4.2.6]), thymidine can then be channeled to DNA biosynthesis [66]. The nucleoside deoxyribosyltransferase gene expression was also highest in the rumen microbes of ACon cattle (Fig. S11), and lactic acid bacteria are reported rich in nucleoside deoxyribosyltransferase [67]. This shows excellent consistency with the enriched lactic acid bacteria in the rumen of ACon cattle. The differential ruminal metabolites detected from A16Ccon vs BT illustrated that L-ornithine upregulation was the most obvious, and its synthetic precursors (L-glutamate and N-acetyl-L-glutamic-acid) and catabolite polyamines (spermidine, 1,3-propanediamine, spermine) levels were also higher in A16Ccon group. These metabolites are mainly involved in arginine biosynthesis and arginine and proline metabolism. L-ornithine is derived from arginine catabolism and de novo biosynthesis. The L-ornithine biosynthetic pathway begins with α-ketoglutarate and can be divided into two stages, the first stage is the synthesis of L-glutamate, and the second stage is the synthesis of ornithine. The conversion of L-glutamate to N-acetyl-L-glutamate is activated by N-acetylglutamate synthase (ArgJ), then through a series of enzymatic reactions to form L-ornithine catalyzed by N-acetylglutamate kinase (ArgB), N-acetyl-gamma-glutamyl-phosphate reductase (ArgC), acetylornithine aminotransferase (ArgD), and acetylornithine deacetylase (ArgE). Polyamines starts from the decarboxylation of L-ornithine to putrescine catalyzed by ornithine decarboxylase then converted to spermidine, 1,3-propanediamine, and spermine. Ruminants express plasma amine oxidase; when spermidine and spermine enter systemic blood circulation, they can be oxidized into aldehyde and hydrogen peroxide by amine oxidase [68]. Aldehydes and hydrogen peroxide are known to cause oxidative stress in eukaryotic cells, and the serum MDA and COR concentration in A16Con cattle was upregulated. Interestingly, a previous study reported that blood COR could promote intestinal polyamine synthesis by enhancing the activity of ornithine decarboxylase (the first rate-limiting enzyme for polyamine synthesis) [69, 70]. This means that the elevated serum COR level induced by polyamine-mediated oxidative stress in A16Con cattle could increase polyamine production. Besides being toxic to the circulatory system, the accumulation of hydrogen peroxide induced by spermidine and spermine might also be harmful to rumen microbes, as that rumen is deficient in catalases to decompose hydrogen peroxide. Intriguingly, adenosine-diphosphate-ribose (ADPR), a highly reactive molecule generated from NAD hydrolysis, may be stimulated by hydrogen peroxide [71], was ranked the third in the upregulated differential metabolites according to the VIP value (Fig. 6B). In bacteria, NAD plays a crucial role in DNA ligation as an energy donor; ADPR concentration can act as a signal of NAD pool consumption [72]. The increased ruminal ADPR level in A16Con might suggest excessive consumption of NAD and impaired bacteria DNA ligation. In addition, ADPR can cause non-enzymatic glycation of proteins and further their inactivation [73]. Together, these studies show that ADPR had a negative effect in rumen metabolism. In this study, correlation analysis shows positive correlation between *Saccharopolyspora rectivirgula* and spermine, spermidine, 1,3-propanediamine, N-acetyl-L-glutamic-acid, N2-acetyl-L-ornithine, L-ornithine, and L-glutamate in the rumen of A16Con cattle (Fig. 8B). A previous study also reported that *Saccharopolyspora* was able to produce spermidine even when they were grown in the synthetic polyamine-free 199 medium [74]. Combined with our aforementioned *Saccharopolyspora rectivirgula* pro-inflammatory bacteria, it can be speculated that *Saccharopolyspora rectivirgula* causes oxidative stress in beef cattle by promoting the release of polyamines into blood, which leads to inflammation. In A16Con vs. BT, in addition to the arginine biosynthesis and arginine and proline metabolism, vitamin B6 metabolism was also the pathway significantly enriched with differential metabolites (Fig. 6B), which included three downregulated metabolites pyridoxamine, 4-pyridoxic acid, and P-salicylic acid, and one upregulated metabolite D-ribulose-5-phosphate (Table S6). Even though D-ribulose-5-phosphate was upregulated, it was also involved in pentose and glucoronate interconversion, pentose phosphate pathway, lipopolysaccharide biosynthesis, methane metabolism etc.; this may suggest that vitamin B6 metabolism in the rumen of A16Con cattle was decreased. Vitamin B6, including six vitamers: pyridoxine, pyridoxal, pyridoxamine, and the phosphate ester form of each, has an essential role in cells as a cofactor in a wide range of biochemical reactions [75] and can also affect the host immune response through both cellular and humoral immunity [76]. Under normal physiological conditions, rumen microbes can synthesize a large amount of vitamin B6, and ruminants do not require dietary vitamin B6 supplementation. Therefore, the downregulated ruminal microbiota vitamin B6 metabolism in A16Con cattle may disrupt host metabolism and immune function. Finally, it is worth noting that the VIP value of adenosine-2',3'-cyclic phosphate (2',3'-cAMP) was ranked last in the downregulated differential metabolites. As a positional isomer of the second messenger cAMP (3',5'-cAMP), 2',3'-cAMP can also act as a second messenger in both eukaryotes and prokaryotes [77]. In pathogenic bacteria (*Escherichia coli*), depletion of 2',3'-cAMP promotes biofilm production, while increasing 2',3'-cAMP concentration resulted in biofilm formation inhibition [77]. Biofilm formation is crucial for the virulence of pathogenic bacteria and is mainly governed by nucleotide metabolism and cyclic dimeric-3',5'-GMP (c-di-GMP) signaling. Perturbation of 2',3'-cyclic nucleotide monophosphate (2',3'-cNMPs) levels modulate the de novo pyrimidine or purine nucleotide biosynthesis which suggests that 2',3'-cNMPs modulate nucleotide pools and can perturb c-di-GMP signaling, which likely mediates the biofilm-associated microbial pathogenesis [77]. Therefore, in the A16Con cattle, the decreased contents of ruminal 2',3'-cAMP, and correlated nucleotide metabolites (cAMP, cGMP, and adenine) provided favorable growth conditions for pathogenic bacteria. Intriguingly, precisely opposite to the A16Con vs. BT (Fig. 6B), ruminal L-ornithine and 2',3'-cAMP were the most downregulated and upregulated differential metabolites in A30Con vs. A16Con, respectively. It is consistent with L-ornithine, and its associated metabolites including spermidine, 1,3-propanediamine, L-glutamate, N-acetyl-L-glutamic acid, L-4-hydroxyglutamate semialdehyde, and N2-acetyl-L-ornithine were downregulated. Likely with 2',3'-cAMP, cytidine 2',3'-cyclic phosphate (2',3'-cyclic CMP), another species in 2',3'-cNMPs pools was also upregulated. At the same time, the levels of cGMP, adenine, and cAMP involved in 2',3'-cNMPs generation were increased. Changes in ruminal metabolites in A30Con cattle compared to A16Con cattle may suggest that, with the decreased of L-ornithine, the oxidative stress induced by L-ornithine catabolites (mainly spermidine and 1,3-propanediamine) got relief; with the increase of 2',3'-cAMP-based 2',3'-cNMPs pools, the pathogenic bacteria biofilm formation was inhibited. This is also consistent with the changes in redox, inflammation, and host immune status.

Comparative serum metabolomics analysis of ACon vs. BT identified 134 differential metabolites. These metabolites were mainly enriched in purine metabolism, D-glutamine and D-glutamate metabolism, alanine, aspartate and glutamate metabolism, arginine and proline metabolism, arginine biosynthesis, etc. These pathways are collectively part of skeletal muscle catabolism and energy metabolism. During long-distance transportation, beef cattle suffer from starvation and skeletal muscle breakdown and release L-glutamine and L-alanine into the blood to support cellular functions [78]; muscle repeatedly and vigorously contracts, decomposes large amounts of adenosine triphosphate (ATP) in muscle and release inosine, hypoxanthine, and xanthine into the blood. In addition, the creatine-phosphocreatine system is crucial for the production of ATP; creatine kinase catalyzes the reversible conversion of phosphocreatine and adenosine diphosphate to form creatine and ATP, respectively. As a result, in the serum of ACon cattle, purine metabolism (inosine, guanine, xanthine, guanosine, and hypoxanthine), L-glutamine, and creatine were higher than those in BT cattle. Within downregulated serum metabolites in ACon vs. BT, 5-hydroxy-6-methoxyindole glucuronide was the most downregulated metabolite according to VIP value. Glucuronide, a key metabolite of glucose, plays a key detoxification role bycombining with a variety of harmful substances in the liver [79]. 5-hydroxy-6-methoxyindole glucuronide is glucuronidation product of 5-hydroxy-6-methoxyindole. Indoles endogenous metabolites are usually produced from tryptophan metabolism [80]. The serum 5-hydroxy-6-methoxyindole glucuronide decreased significantly in ACon vs. BT, suggesting that the detoxification/glucuronidation weakened in the ACon cattle. Moreover, we detected PC species, LysoPC species, and phosphocholine imbalances in all ACon vs. BT, A16Con vs. BT, and A30Con vs. A16Con, possibly reflecting the cellular response to stress and activation. PC, known as anti-inflammatory or antioxidant phospholipids, participates in membrane structure formation and cell signaling [81]. LysoPC is a bioactive lysophospholipid produced by hydrolysis of PC by phospholipase A2 in the Lands cycle [82]. The role of LysoPC in systemic inflammation is controversial; both pro-inflammatory and anti-inflammatory effects have been reported. Someone summed that sepsis, human diabetes mellitus, and atherosclerosis present low levels of LysoPC; systemic treatment with LysoPC has shown therapeutic efficacy in inflammatory models, including sepsis and cerebral ischemia [83]. In dairy cattle, heat stress significantly reduced the milk LysoPC, which appears to be a lipid marker for heat stress in dairy cattle [84]. They explained that the reduction of LysoPC may be attributed to the inhibited formation of choline/phosphocholine in the biosynthesis pathways under heat stress [84]. Choline kinase catalyzes the production of phosphocholine from choline and ATP, a rate-limiting step in PC synthesis. ATP depletion under stressful conditions may block phosphocholine biosynthesis, thereby inhibiting PC production. In the present study, the phosphocholine was also downregulated in ACon vs. BT. It is inferred that the decrease of PC and LysoPC species in ACon vs. BT may be due to the transport stress in this study. As the blood sample of ACon cattle was sampled 1 day after arrival, the up-stream steps in the PC and LysoPC biosynthesis were not fully recovering from the stress. Surprisingly, the serum differential metabolites in both A16Con vs. BT and A30Con vs. A16Con were almost all the PC and LysoPC. However, they only downregulated metabolites in A16Con vs. BT and only upregulated in A30Con vs A16Con. To explain this phenomenon, we first briefly introduce the synthesis process of PC. Two pathways are responsible for the de novo synthesis of PC, namely the phosphatidylethanolamine-N-methyltransferase (PEMT) and the Kennedy pathways. PEMT performs three sequential methylation reactions to convert phosphatidylethanolamine (PE) into PC, using S-adenosylmethionine as a methyl donor [85]. In the Kennedy pathway, serine is decarboxylated to produce ethanolamine, which obtains three methyl groups from S-adenosylmethionine to generate choline. It can be seen that PC synthesis is inseparable from S-adenosylmethionine as a methyl donor. While, as the most significantly changed metabolites in the rumen of A16Con and A30Con cattle, L-ornithine can be decarboxylated by ornithine decarboxylase to generate putrescine, which further reacts with S-adenosylmethionine to generate spermidine and spermine [86]. Since polyamines compete with PC for the biosynthetic precursor S-adenosylmethionine, it can be speculated that high polyamine production from L-ornithine may inhibit PC biosynthesis in the A16Con cattle; in turn, the decreased ruminal L-ornithine and polyamines accompanied by increased serum PC and LysoPC appearing in the A30Con cattle. Finally, it should be mentioned that the ability of rumen protozoa to degrade arginine is considered much slower than bacteria; alsorumen protozoa showed lower degradation activity of citrulline to ornithine than rumen bacteria. Therefore, the decrease of L-ornithine concentration in the rumen of A30Con cattle may be partially associated with an increased ratio of ruminal protozoa [87].

## Conclusion

**Fig. 9:**
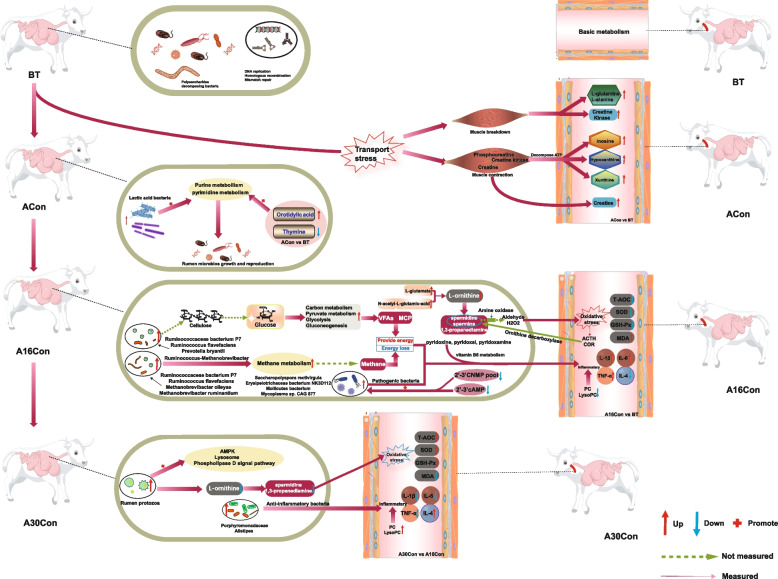
Full text of the overview summary. Rumen microbial species, rumen microorganisms’ function, rumen metabolites, and serum metabolites were analyzed among the four groups. The top five differentially abundant microbial species and the main differential KEGG function were demonstrated (LDA score of > 2.0 and *P* value of < 0.05). The rumen and serum metabolism were mainly analyzed in ACon vs. BT, A16Con vs BT and A30Con vs BT. Metabolites were selected by VIP > 1 and *P* < 0.05. Before transport, the most bacteria were involved in polysaccharides digestion. At day 1 after-transport, serum upregulated metabolites were mainly associated with skeletal muscle catabolism and energy metabolism. At day 4 after transport, rumen microbes were in rapid reproduction and recovered by enriched pyrimidine metabolism and purine metabolism within rumen microbial functions, and higher orotidylic acid concentration from rumen microbial metabolites at this stage. At day 16 after-transport, the energy metabolism was enhanced and ruminal contents of MCP and VFAs were highest, the energy loss induced by higher methane yields and the pathogenic bacteria together caused inflammation and oxidative stress in the A16Con cattle. At this time, the most upregulated ruminal L-ornithine produces more catabolites polyamines, which cause oxidative stress to rumen microbes and their host; the most downregulated ruminal 2',3'-cAMP provided favorable growth conditions for pathogenic bacteria; and the downregulated ruminal vitamin B6 metabolism and serum PC/LysoPC disrupt immune function and inflammation reaction. At day 30 post-transport, with the decrease of L-ornithine and the increase of protozoa, the oxidative stress is relieved, and the increase of anti-inflammatory bacteria can alleviate the body’s inflammatory reaction

This study identified that the rumen microbial composition, functions, and metabolites, together with host metabolism and health status of newly received cattle during the receiving period, had significant differences. The inflammation and oxidative stress in newly received cattle were most severe at day 16 after transport, and relieved at day 30 after transport. The mechanism was related to rumen microbiota, metabolism, and host metabolism. Although energy metabolism (glycolysis/gluconeogenesis, pyruvate metabolism) was enhanced and ruminal contents of MCP and VFAs were highest, the energy loss induced by higher methane yields (*Methanobrevibacter*) and pathogenic bacteria (*Saccharopolyspora rectivirgula*) together caused inflammation and oxidative stress in the A16Con cattle. At this time, the most upregulated ruminal L-ornithine produced more catabolites polyamines, which caused oxidative stress to rumen microbes and their host; the downregulated ruminal vitamin B6 metabolism and serum PC/LysoPC (compete with polyamines for the biosynthetic precursor S-adenosylmethionine) disrupt immune function and inflammation reaction. These rumen and serum metabolites together further explain the host’s health status (Fig. 9). This study provides new ideas for regulating the health and performance of newly received cattle during the receiving period. The critical point is to manage the newly received cattle about day 16 after transport, specifically to inhibit the production of methane and polyamines, and the reproduction of harmful bacteria in the rumen, therefore improving the immunity and performance of newly received cattle.

## Methods

### Animal treatments and experimental diets

Thirty-two 6-month-old male Chinese Simmental crossbred cattle, raised under identical feeding and environmental conditions, with similar genetic backgrounds, were used in this study. They were allotted to four treatments and ear tagged; each group consisted of eight cattle. Group 1 as control group with general management. Groups 2, 3, and 4 with drinking water creatine pyruvate (CrPyr, 30 g/day per cattle) supplementation for various days, which were the day of departure, the day of departure + 15 days after transport, and the day of departure + 30 days after transport, respectively. CrPyr is a new multifunctional nutrient, our previous studies have been shown that CrPyr could relieve the heat and transport stress of beef cattle [9, 88]. Although the experiment included four treatment groups, each group sampled at four different time points included three MetaOmics analyses in Group 1 (control group) and Group 4. All the results are presented in one paper may result in the paper is too long. This article mainly focuses on rumen metagenomics, metabonomics, and blood metabonomics of beef cattle before transport (1 day) and after transport (at day 1 or 4, 16 and 30) based on the control group; the effects of pyruvate creatine were not introduced in detail in the present study.

These 32 experimental cattle together with 68 other cattle were transported by the double layer car, length 13.5 m, width 2.3 m, and height 4.2 m (Zhumadian CIMC HuaJun Vehicle Co., Ltd, China), equipped with quilt mattress and covering. The 32 experimental cattle were located on the upper level, the rest of the cattle were randomly allocated, with 50 cattle on each double-deck, with an average of 0.63 m^2^ per cattle. Cattle transport from the Live Animals and Forage Trading Market in Altay city in Xinjiang Province to Yuzhou city in Henan Province at 20.00 h on September 13, 2021, and arrived at the Yuzhou Longyue Animal Husbandry Co., Ltd, Xuchang, Hehan, at 15.00 h on September 16, 2021, which represents a travel time of 67 h and a distance of 3450 km on highway and city roads at a maximum speed of 70 km/h.

Cattle were deprived of water during transportation, while they could eat some wheat straw, which was placed on the sides of the cart in the form of straw bundles when loading. Before transport, the ratio of concentrate and wheat straw in diet was 30:70. The concentrate was purchased from commercial company, including crude protein ≥ 17%, crude fiber ≤ 10%, crude ash ≤ 9%, moisture ≤ 14%, total phosphorus ≥ 0.4%, calcium 0.5–1.2%, and salt 0.8–1.5%. After arrival, the experimental cattle were tethered in the same beef cattle cowshed in grouping order. In the first 2 days, cattle were mainly fed with wheat straw, and in subsequent experiments, cattle were fed with total mixed ration (TMR). The composition and nutrient levels of the TMR are presented in Table S1, and the TMR was provided twice daily. Drinking water intake of beef cattle was strictly controlled in the first 5 days after arrival. The water temperature was about 37 °C, given three times a day with about 3 L each time, and in subsequent experiments, normal temperature water was available freely. In addition, in the first five days after arrival, newly received cattle were supplemented with the probiotics and probiotic metabolites (mainly *Bacillus subtilis*, *Saccharomyces cerevisiaeits*, and their metabolites) obtained from Shandong Helai Biotechnology Co., Ltd (Shandong, China) in the drinking water (10 g/L).

### Sample collection and measurements

Blood and rumen fluid were sampled at day 1 before transport, and then at days 1/4, 16, and 30 after transport in the morning before feeding. The newly received cattle in control group were divided into 4 subgroups based on the sampling time: BT = day 1 before transport, simultaneous collection of blood and rumen fluid; ACon = day 1 or 4 after transport, collection of blood at day 1 post-transport, collection of rumen fluid at day 4 post-transport; A16Con = day 16 post-transport, simultaneous collection of blood and rumen fluid; A30Con = day 30 post-transport, simultaneous collection of blood and rumen fluid. Ruminal fluid was collected using an oral stomach tube. The device was cleaned thoroughly between sample collections using warm and clean water, and the first 50 mL of rumen fluid was discarded to minimize saliva contamination. Then, 50 mL of rumen fluid sample collected from each animal was immediately measured for pH using a portable pH meter (HANNA Instruments, Cluj-Napoca, Romania). After that, the sample was divided into sterilized portions, immediately frozen with liquid nitrogen, and stored at − 80 °C until further analysis. Blood sample (10 mL) was taken from the jugular vein of cattle into evacuated nonanticoagulative tubes. The blood samples were centrifuged at 3000* g* (10 min, 4 °C) to obtain serum samples, and then stored at − 80 °C to measure indices and metabolome profiles.

### Chemical analyses

The VFA concentrations in the rumen fluid samples were determined using gas chromatography (Shimadzu GC-2014, Japan) equipped with a capillary column (Stabilwax, Restek, Bellefonte, PA, USA). The NH_3_-N concentration was measured using a TU-1901 spectrophotometer (Beijing Purkinje General Instrument Co. Ltd., China) according to a method described by Broderick and Kang [89]. MCP production was determined according to the method of Coomassie Brilliant Blue [90]. Serum lactate dehydrogenase (LDH), creatine kinase (CK), cortisol (COR), adreno cortico tropic hormone (ACTH), total antioxidant capacity (T-AOC), superoxide dismutase (SOD), glutathione peroxidase (GSH-PX), malondialdehyde (MDA), IgA, IgG, IgM, interleukin-1β (IL-1β), IL-4, IL-6, and tumor necrosis factor-α (TNF-α) were determined using commercial kits (Nanjing Jiancheng Bioengineering Institute, Nanjing, China).

### Rumen metagenomics

#### DNA extraction, library construction, and metagenomic sequencing

Total genomic DNA was extracted from rumen fluid using the FastDNA™ Spin Kit for Soil (MP Biomedicals, USA) according to the manufacturer’s instructions. Concentration and purity of extracted DNA was determined with TBS-380 and NanoDrop2000, respectively. DNA extract quality was checked on 1% agarose gel. DNA extract was fragmented to an average size of about 400 bp using Covaris M220 (Gene Company Limited, China) for paired-end library construction. Paired-end library was constructed using NEXTflex™ Rapid DNA-Seq (Bioo Scientific, Austin, TX, USA). Adapters containing the full complement of sequencing primer hybridization sites were ligated to the blunt-end of fragments. Paired-end sequencing was performed on Illumina NovaSeq 6000 (Illumina Inc., San Diego, CA, USA) with PE150 at Majorbio Bio-Pharm Technology Co., Ltd. (Shanghai, China) using NovaSeq Reagent Kits according to the manufacturer’s instructions (www.illumina.com). Sequence data associated with this project have been deposited in the NCBI Short Read Archive database (BioProject ID: PRJNA943223).

#### Sequence quality control and genome assembly

The raw reads from metagenome sequencing were used to generate clean reads by removing adaptor sequences, trimming and removing low-quality reads (reads with N bases, a minimum length threshold of 50 bp and a minimum quality threshold of 20) using the fastp [91] (https://github.com/OpenGene/fastp, version 0.20.0) on the free online platform of Majorbio Cloud Platform (cloud.majorbio.com). Reads were aligned to the Bos taurus (cattle) genome by BWA [92] (http://bio-bwa.sourceforge.net, version 0.7.9a) and any hit associated with the reads and their mated reads were removed. These high-quality reads were then assembled to contigs using MEGAHIT [93] (parameters: kmer_min = 47, kmer_max = 97, step = 10) (https://github.com/voutcn/megahit, version 1.1.2), which makes use of succinct de Bruijn graphs. Contigs with the length being or over 300 bp were selected as the final assembling result.

#### Gene prediction, taxonomy, and functional annotation

Open reading frames (ORFs) in contigs were identified using MetaGene [94] (http://metagene.cb.k.u-tokyo.ac.jp/). The predicted ORFs with length being or over 100 bp were retrieved and translated into amino acid sequences using the NCBI translation table (http://www.ncbi.nlm.nih.gov/Taxonomy/taxonomyhome.html/index.cgi?chapter=tgencodes#SG1).

A non-redundant gene catalog was constructed using CD-HIT [95] (http://www.bioinformatics.org/cd-hit/, version 4.6.1) with 90% sequence identity and 90% coverage. Reads after quality control were mapped to the non-redundant gene catalog with 95% identity using SOAPaligner [96] (http://soap.genomics.org.cn/, version 2.21), and gene abundance in each sample were evaluated.

Representative sequences of non-redundant gene catalog were annotated based on the NCBI NR database using blastp as implemented in DIAMOND v0.9.19 with e-value cutoff of 1e^−5^ using Diamond [97] (http://www.diamondsearch.org/index.php, version 0.8.35) for taxonomic annotations. The KEGG annotation was conducted using Diamond [97] (http://www.diamondsearch.org/index.php, version 0.8.35) against the Kyoto Encyclopedia of Genes and Genomes database (http://www.genome.jp/keeg/, version 94.2) with an e-value cutoff of 1e^−5^. Carbohydrate-active enzyme annotation was conducted using hmmscan (http://hmmer.janelia.org/search/hmmscan) against CAZy database (http://www.cazy.org/) with an e-value cutoff of 1e^−5^.

### Rumen and serum metabolomics

The rumen and serum samples were analyzed using the LC–MS platform (Thermo, UHPLC -Q Exactive HF-X). Sequence data associated with this project have been deposited in MetaboLights (ID: MTBLS7520).

### Metabolite extraction

Rumen fluid (30 mg) was accurately weighed and transferred into centrifuge tube (2 mL) with a grinding bead (6 mm diameter). The metabolites extracted using a 400 µL methanol:water (4:1, v/v) solution contains 0.02 mg/mL internal standard (L-2-chlorophenylalanine). The mixture was allowed to settle at − 10 °C and treated by high-throughput tissue crusher Wonbio-96c (Shanghai wanbo biotechnology co., LTD) at 50 Hz for 6 min, then followed by ultrasound at 40 kHz for 30 min at 5 °C. The samples were placed at − 20 °C for 30 min to precipitate proteins. After centrifugation at 13,000* g* at 4 °C for 15 min, the supernatant was carefully transferred to sample vials for LC–MS/MS analysis.

Serum (100 µL) was accurately transferred into a centrifuge tube (1.5 mL), after addition of 400 µL of methanol:acetonitrile (1:1, v/v) solution contains 0.02 mg/mL internal standard (L-2-chlorophenylalanine), each sample was vortexed for 30 s and ultrasound at 40 kHz for 30 min at 5 °C. The samples were placed at − 20 °C for 30 min to precipitate proteins. After centrifugation at 13,000* g* at 4 °C for 15 min, the supernatant was carefully transferred and dried by nitrogen, and then dissolved in 120 µL of acetonitrile:water (1:1, v/v) solution. Following, the sample was vortexed for 30 s and ultrasound at 40 kHz for 5 min at 5 °C. After centrifugation at 13,000* g* at 4 °C for 15 min, the supernatant was carefully transferred to sample vials for LC–MS/MS analysis.

### LC–MS/MS analysis

Chromatographic separation of the metabolites was performed using an ACQUITY BEH C18 column (100 mm × 2.1 mm i.d., 1.8 µm; Waters, Milford, USA). The mobile phases consisted of 0.1% formic acid in water:acetonitrile (95:5, v/v) (solvent A) and 0.1% formic acid in acetonitrile:isopropanol:water (47.5:47.5:5, v/v) (solvent B). The sample injection volume was 2 µL and the column temperature was maintained at 40 °C. During the period of analysis, all these samples were stored at 4 °C. Spectrometer equipped with an electrospray ionization (ESI) source operating in either positive or negative ion mode. The optimal conditions were set as follows: scan type, 70–1050 m/z; sheath gas flow rate, 50 arb; Aux gas flow rate, 13 arb; heater temperature, 425 °C; capillary temperature, 325 °C; ion-spray voltage floating (ISVF), − 3500 V in negative mode and 3500 V in positive mode, respectively; normalized collision energy, 20–40-60 V rolling for MS/MS. Data acquisition was performed with the data-dependent acquisition (DDA) mode. To obtain information regarding system repeatability, quality control (QC) samples prepared by mixing all rumen fluid/serum extraction aliquots were injected at regular intervals (every 6 samples) throughout the analytical run.

### Metabolomics data analysis

The raw data were imported into the Progenesis QI 2.3 (Waters Corporation, Milford, USA) for peak detection and alignment. The preprocessing results generated a data matrix that consisted of the retention time (RT), mass-to-charge ratio (m/z) values, and peak intensity. Metabolic features detected at least 50% in any set of samples were retained. After filtering, minimum metabolite values were imputed for specific samples in which the metabolite levels fell below the lower limit of quantitation and each Metabolic features were normalized by sum. The internal standard was used for data QC (reproducibility), metabolic features which the relative standard deviation (RSD) of QC > 30% were discarded. Following normalization procedures and imputation, statistical analysis was performed on log transformed data to identify significant differences in metabolite levels between comparable groups. Mass spectra of these metabolic features were identified by using the accurate mass, MS/MS fragments spectra, and isotope ratio difference with searching in reliable biochemical databases as Human metabolome database (HMDB) (http://www.hmdb.ca/) and Metlin database (https://metlin.scripps.edu/). Concretely, the mass tolerance between the measured m/z values and the exact mass of the components of interest was ± 10 ppm. For metabolites having MS/MS confirmation, only the ones with MS/MS fragments score above 30 were considered as confidently identified. Otherwise, metabolites had only tentative assignments.

### Multivariate statistical analysis

A multivariate statistical analysis was performed using ropls (Version1.6.2, http://bioconductor.org/packages/release/bioc/html/ropls.html) R package from Bioconductor on Majorbio Cloud Platform (https://cloud.majorbio.com). Principle component analysis (PCA) using an unsupervised method was applied to obtain an overview of the metabolic data, general clustering, trends, or outliers were visualized. All of the metabolite variables were scaled to unit-variances prior to conducting the PCA. Orthogonal partial least squares discriminate analysis (OPLS-DA) was used for statistical analysis to determine global metabolic changes between comparable groups. All of the metabolite variables were scaled to Pareto Scaling prior to conducting the OPLS-DA. The model validity was evaluated from model parameters R2 and Q2, which provide information for the interpretability and predictability, respectively, of the model and avoid the risk of over-fitting. Variable importance in the projection (VIP) were calculated in OPLS-DA model.* P* values were estimated with paired Student’s *t* test on single dimensional statistical analysis.

### Differential metabolites analysis

Statistically significant among groups were selected with VIP value more than 1 and *P* value less than 0.05. In total, 642 rumen metabolites and 503 serum metabolites were identified. Differential metabolites among two groups were summarized and mapped into their biochemical pathways through metabolic enrichment and pathway analysis based on database search (KEGG, http://www.genome.jp/kegg/). These metabolites can be classified according to the pathways they involved or the functions they performed. Enrichment analysis was usually to analyze a group of metabolites in a function node whether it appears or not. The principle was that the annotation analysis of a single metabolite develops into an annotation analysis of a group of metabolites. scipy.stats (Python packages) (https://docs.scipy.org/doc/scipy/) was exploited to identify statistically significantly enriched pathway using Fisher’s exact test.

## Statistical analysis

All the data mentioned above were analyzed by one-way ANOVA with SPSS (version 17.0, IBM, Armonk, NY, USA). The significant difference among treatments was compared by Tukey’s multiple range test. A *P* value < 0.05 was considered statistically significant. The results were presented as the mean values and the standard error of the mean. Correlation analysis between rumen bacteria, rumen fermentation characteristic, serum indicators, rumen metabolome, and serum metabolome was performed using Spearman’s rank correlation and visualized in a heatmap format using R (Version 3.3.1, R Core Team, Vienna, Austria) “pheatmap package”. The absolute value of correlation coefficients (|*r*|> 0.50 and *P* < 0.05) was considered as significant.

### Supplementary Information

**Additional file 1: Table S1.** Composition and nutrient levels of experimental diet (air-dry basis, %).

**Additional file 2: Table S2.** Summary of sequence data generated from newly received cattle at 1 day before transportation, and then at day 4, day 16, day 30 after transportation.

**Additional file 3: Table S3.** Composition of metabolic pathways based on the first-level and second-level functions in the KEGG.

**Additional file 4: Table S4.** Detailed results of differencial metabolic three-level pathways pathways based on the LEfSe analysis.

**Additional file 5: Table S5.** Composition of CAZymes based on the class-level and family-level enzymes.

**Additional file 6: Table S6. **Detailed differential rumen metabolites identified from the pairwise comparisons among the four groups.

**Additional file 7: Table S7. **Detailed differential serum metabolites identified from the pairwise comparisons among the four groups.

**Additional file 8: Fig. S1. **Profiles of rumen microbial composition of newly received cattle.

**Additional file 9: Fig. S2. **Microbial compositional profiles of (A) Eukaryota and (B) Viruses of newly received cattle rumen samples visualized using principal-coordinate analysis (PCoA).

**Additional file 10: Fig. S3. **Comparison of bacterial phyla and genera. Bacterial phyla (A) and genera (B) were tested by Kruskal-Wallis H test.

**Additional file 11: Fig. S4. **Comparison of archaeal phyla and genera. Archaeal phyla (A) and genera (B) were tested by Kruskal-Wallis H test.

**Additional file 12: Fig. S5.** Differential CAZymes among BT, ACon, A16Con, and A30Con cattle.

**Additional file 13: Fig. S6. **Rumen metabolites of BT, ACon, A16Con, and A30Con cattle categorized according to HMDB (A) and comparison based on OPLS-DA (B).

**Additional file 14: Fig. S7. **Rumen metabolome of A30Con vs BT, A16Con vs ACon, and A30Con vs ACon.

**Additional file 15: Fig. S8. **Serum metabolites of BT, ACon, A16Con, and A30Con cattle categorized according to HMDB (A) and comparison based on PLS-DA (B).

**Additional file 16: Fig. S9. **Serum metabolome of A30Con vs BT, A16Con vs ACon, and A30Con vs ACon.

**Additional file 17: Fig. S10. **Metabolic pathway difference-in-difference analysis on the DNA replication based on metagenomics data.

**Additional file 18: Fig. S11. **Metabolic pathway difference-in-difference analysis on the pyrimidine based on metagenomics data.

## Data Availability

The datasets analyzed during the current study are available from the corresponding author on reasonable request.

## Abbreviations

ACTH: Adreno cortical tropic hormone
CAZymes: Carbohydrate-active enzymes
CK: Creatine kinase
COR: Cortisol
GSH-PX: Glutathione peroxidase
IL-1β: Interleukin-1β
KEGG: Kyoto Encyclopedia of Genes and Genomes
LDH: Lactate dehydrogenase
LEfse: Linear discriminant analysis effect size
LysoPC: Lysophosphatidylcholine
MCP: Microbial crude protein
MDA: Malondialdehyde
PC: Phosphatidylcholine
SOD: Superoxide dismutase
T-AOC: Total antioxidant capacity
TNF-α: Tumor necrosis factor alpha
VFA: Volatile fatty acid
VIP: Variable importance in the projection
