# Supplementary material for: Revealing the developmental characterization of rumen microbiome and its host in newly received cattle during receiving period contributes to formulating precise nutritional strategies
Source: Microbiome. 2023 Nov 3;11:238. doi: 10.1186/s40168-023-01682-z (PMC10623857; doi:10.1186/s40168-023-01682-z)
Supplement: Supplementary file 2 — Additional file 1: Table S1. Composition and nutrient levels of experimental diet (air-dry basis, %). [file 40168_2023_1682_MOESM1_ESM.docx]

**Table S1** Composition and nutrient levels of experimental diet (air-dry basis, %)

| Ingredients | Content | Nutrient levels | Content |
| --- | --- | --- | --- |
| Wheat straw | 7.46 | Dry matter | 87.10 |
| Corn silage | 59.77 | Crude protein | 11.22 |
| Bean dregs | 23.41 | Crude fat | 3.77 |
| Concentrate^1^ | 9.37 | Neutral detergent fiber | 39.00 |
| Total | 100 | Acid detergent fiber | 17.84 |
|  |  | Ash | 6.14 |

^1^The concentrate (product no. Q/JJZZ 001-2016) purchased from Gail (Henan, China), including crude protein ≥ 19%, ash ≤ 15%, total phosphorus ≥ 0.3%, water ≤ 14%, crude fiber ≤ 15%, calcium 0.3-2%, sodium chloride 0.2-2.5%, and lysine ≥ 0.4%.
