# Supplementary material for: Revealing the developmental characterization of rumen microbiome and its host in newly received cattle during receiving period contributes to formulating precise nutritional strategies
Source: Microbiome. 2023 Nov 3;11:238. doi: 10.1186/s40168-023-01682-z (PMC10623857; doi:10.1186/s40168-023-01682-z)
Supplement: Supplementary file 3 — Additional file 2: Table S2. Summary of sequence data generated from newly received cattle at 1 day before transportation, and then at day 4, day 16, day 30 after transportation. [file 40168_2023_1682_MOESM2_ESM.docx]

**Table S2** Summary of sequence data generated from newly received cattle at 1 day before transportation, and then at day 4, day 16, day 30 after transportation

| Samples | Raw reads | Clean reads | Optimized reads | Contigs | N50(bp) | ORFs |
| --- | --- | --- | --- | --- | --- | --- |
| BT_1 | 76785276 | 74904512 | 57177680 | 673823 | 937 | 988249 |
| BT_2 | 71339008 | 69725264 | 55820198 | 884201 | 666 | 1133696 |
| BT_3 | 73756828 | 72144482 | 58470012 | 671247 | 897 | 943799 |
| BT_4 | 68778802 | 66981604 | 55368512 | 698595 | 984 | 1017411 |
| BT_5 | 98342290 | 96099706 | 74785972 | 1165413 | 706 | 1523256 |
| BT_6 | 74116514 | 71581886 | 57861862 | 713719 | 865 | 1001626 |
| ACon_1 | 73927688 | 72203558 | 59637956 | 903361 | 748 | 1213900 |
| ACon_2 | 87976672 | 85944160 | 71666686 | 880245 | 771 | 1183178 |
| ACon_3 | 73104192 | 71178774 | 60674688 | 662876 | 871 | 936052 |
| ACon_4 | 84042714 | 82339964 | 59964580 | 801481 | 743 | 1092801 |
| ACon_5 | 74721196 | 72608640 | 56934528 | 790775 | 736 | 1072665 |
| ACon_6 | 83761968 | 82130818 | 66539806 | 942037 | 747 | 1264203 |
| A16Con_1 | 89851572 | 87347916 | 70335446 | 902462 | 790 | 1217259 |
| A16Con_2 | 80066226 | 77947572 | 63716684 | 1000162 | 704 | 1302705 |
| A16Con_3 | 75094734 | 72876894 | 58389718 | 894685 | 745 | 1186914 |
| A16Con_4 | 82882468 | 81228910 | 65986390 | 953910 | 732 | 1253071 |
| A16Con_5 | 76458934 | 74720644 | 61046206 | 813352 | 721 | 1079205 |
| A16Con_6 | 87235908 | 85516602 | 70061234 | 924885 | 767 | 1238879 |
| A30Con_1 | 73605512 | 71709030 | 59775906 | 911636 | 698 | 1188961 |
| A30Con_2 | 72203088 | 70244940 | 57891768 | 839891 | 683 | 1085607 |
| A30Con_3 | 76625800 | 74941916 | 56794074 | 907306 | 678 | 1140162 |
| A30Con_4 | 80622634 | 78847784 | 62518486 | 937035 | 733 | 1220597 |
| A30Con_5 | 75935782 | 74455308 | 51907094 | 820807 | 624 | 994457 |
| A30Con_6 | 69661374 | 68129398 | 56031486 | 788038 | 791 | 1057866 |
| Total reads | 1.881E+09 | 1835810282 | 1.469E+09 | 20481942 | 18337 | 27336519 |
| Mean | 78370716 | 76492095.1 | 61223207 | 853414 | 764 | 1139021 |
| SD | 7056864 | 6973948 | 5734137 | 115914 | 86 | 131401 |
| SEM | 1088897 | 1076103 | 884796 | 17886 | 13 | 20276 |
