# Supplementary material for: Revealing the developmental characterization of rumen microbiome and its host in newly received cattle during receiving period contributes to formulating precise nutritional strategies
Source: Microbiome. 2023 Nov 3;11:238. doi: 10.1186/s40168-023-01682-z (PMC10623857; doi:10.1186/s40168-023-01682-z)
Supplement: Supplementary file 10 — Additional file 9: Fig. S2. Microbial compositional profiles of (A) Eukaryota and (B) Viruses of newly received cattle rumen samples visualized using principal-coordinate analysis (PCoA). [file 40168_2023_1682_MOESM9_ESM.pdf]

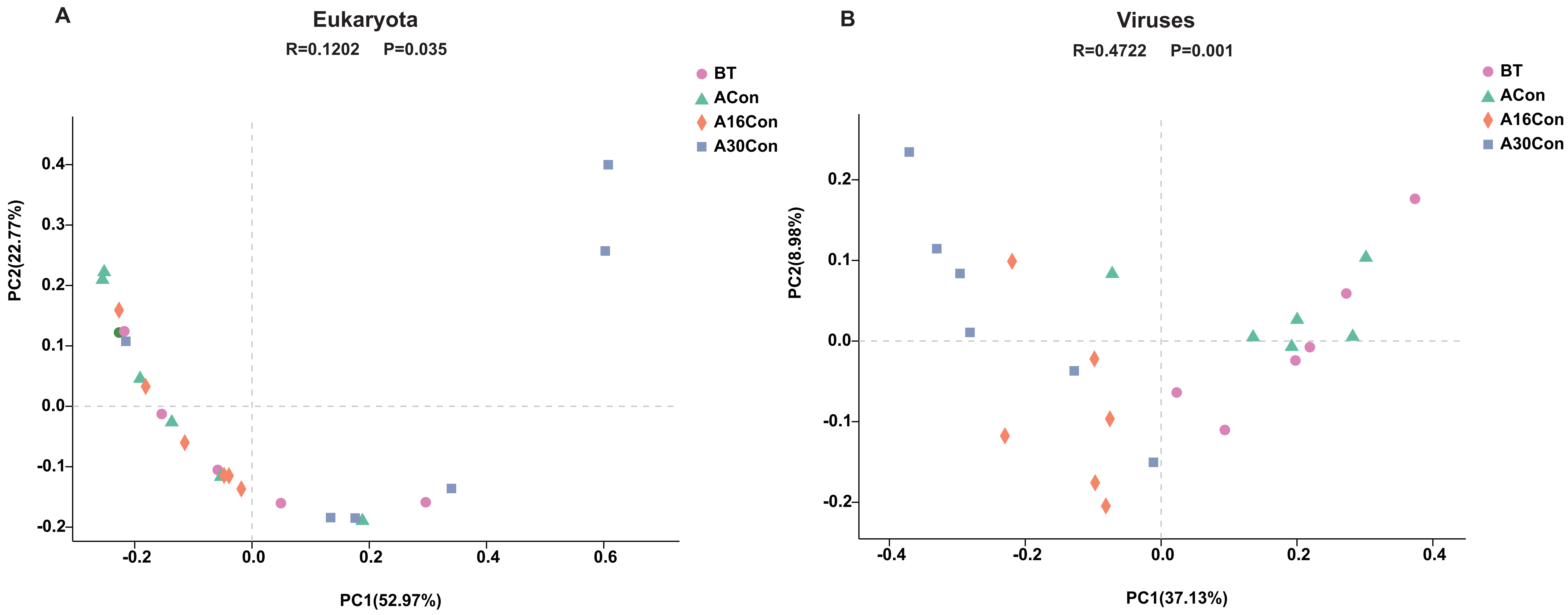

Fig. S2 Microbial compositional profiles of (A) Eukaryota and (B) Viruses of newly received cattle rumen samples visualized using principal-coordinate analysis (PCoA)
