# Supplementary material for: Revealing the developmental characterization of rumen microbiome and its host in newly received cattle during receiving period contributes to formulating precise nutritional strategies
Source: Microbiome. 2023 Nov 3;11:238. doi: 10.1186/s40168-023-01682-z (PMC10623857; doi:10.1186/s40168-023-01682-z)
Supplement: Supplementary file 11 — Additional file 10: Fig. S3. Comparison of bacterial phyla and genera. Bacterial phyla (A) and genera (B) were tested by Kruskal-Wallis H test. [file 40168_2023_1682_MOESM10_ESM.pdf]

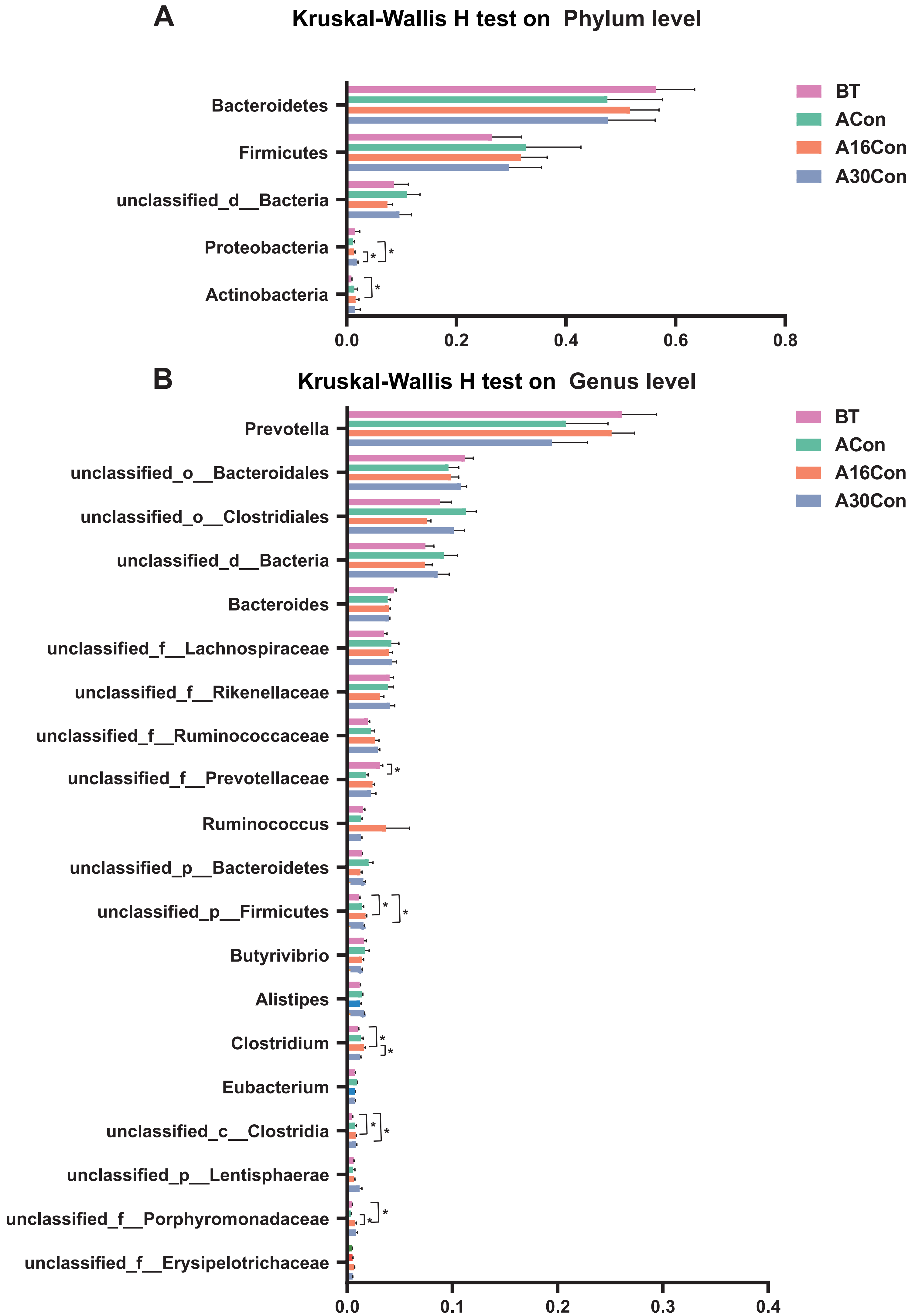

Fig. S3 Comparison of bacterial phyla and genera. Bacterial phyla (A) and genera (B) were tested by Kruskal-Wallis H test
