# Supplementary figures and images for: Revealing the developmental characterization of rumen microbiome and its host in newly received cattle during receiving period contributes to formulating precise nutritional strategies
Source: Microbiome. 2023 Nov 3;11:238. doi: 10.1186/s40168-023-01682-z (PMC10623857; doi:10.1186/s40168-023-01682-z)

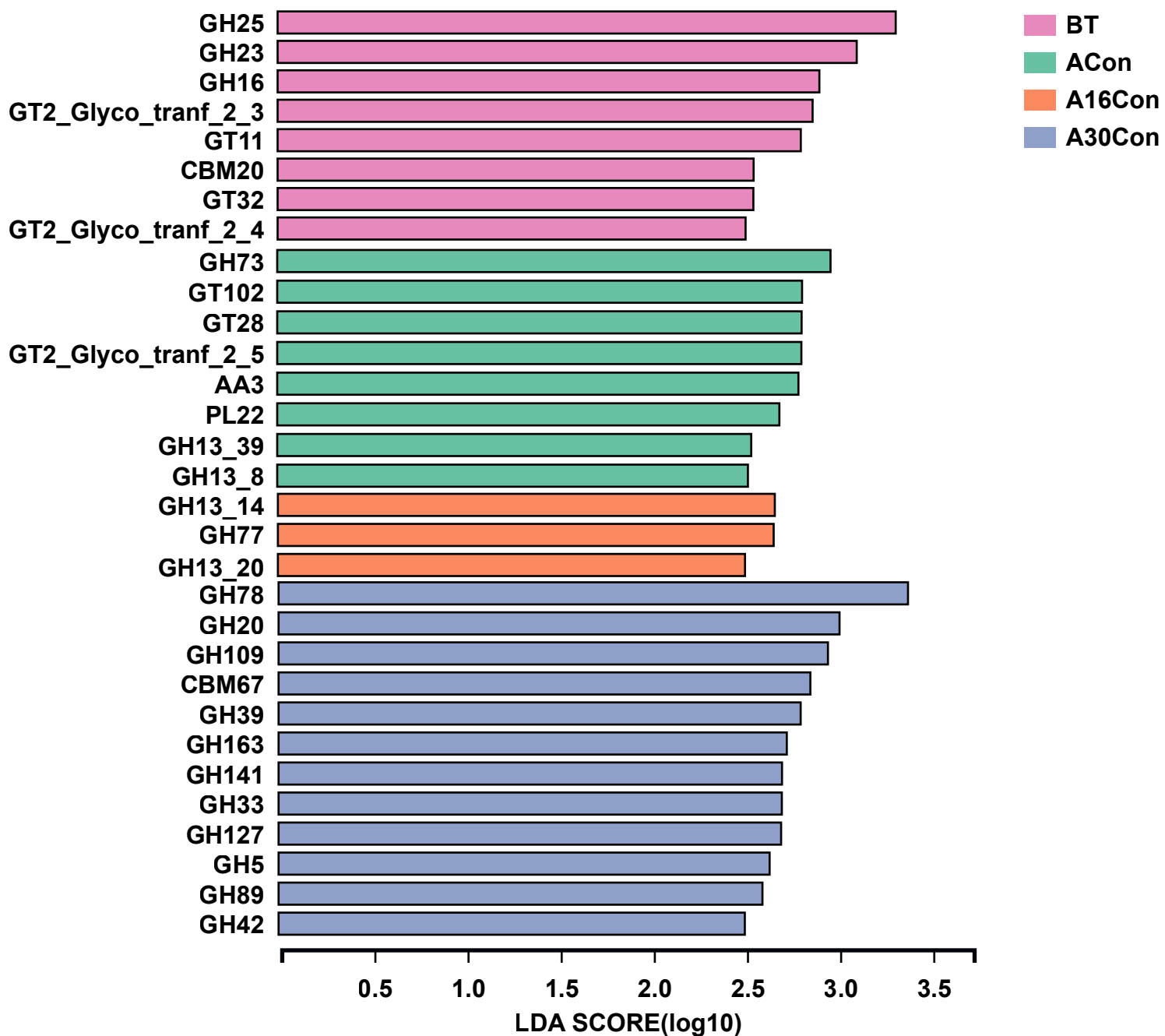

**Fig. S5** Differential CAZymes among BT, ACon, A16Con, and A30Con cattle

Supplement: Supplementary file 13 — Additional file 12: Fig. S5. Differential CAZymes among BT, ACon, A16Con, and A30Con cattle. [file 40168_2023_1682_MOESM12_ESM.pdf]

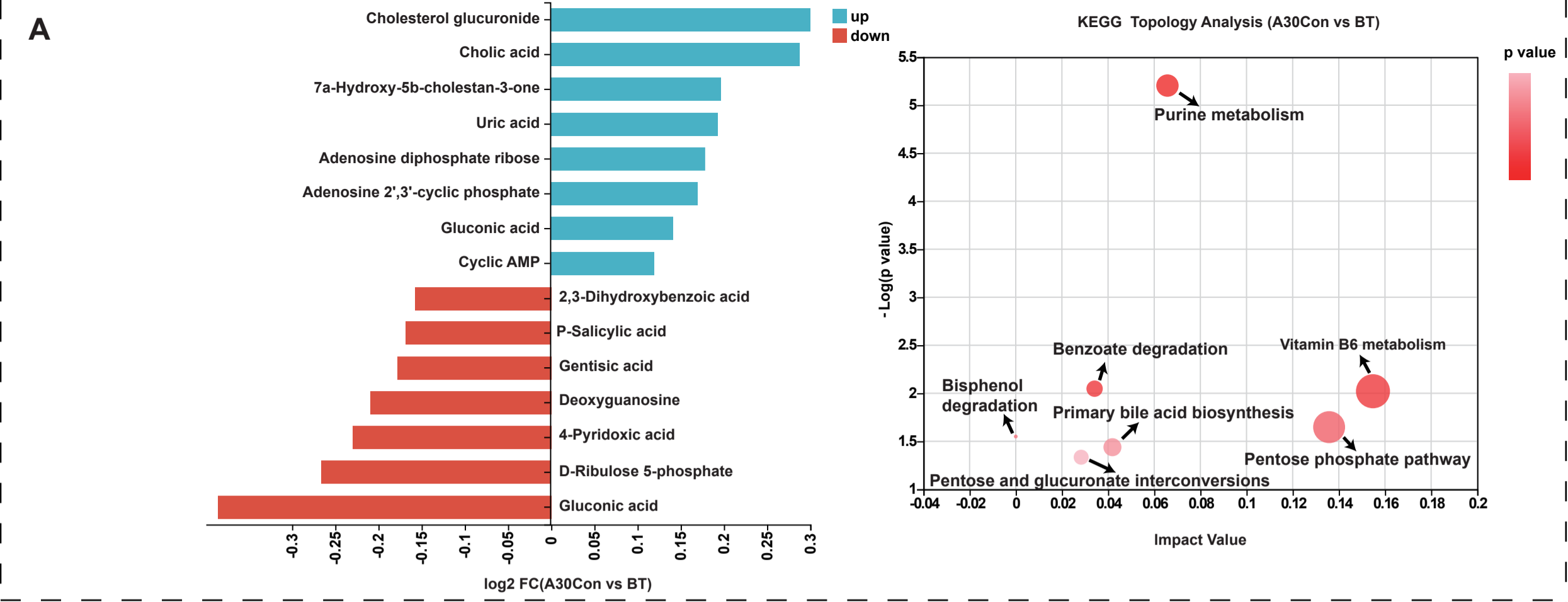

Supplement: Supplementary file 15 — Additional file 14: Fig. S7. Rumen metabolome of A30Con vs BT, A16Con vs ACon, and A30Con vs ACon. [file 40168_2023_1682_MOESM14_ESM.pdf]

A

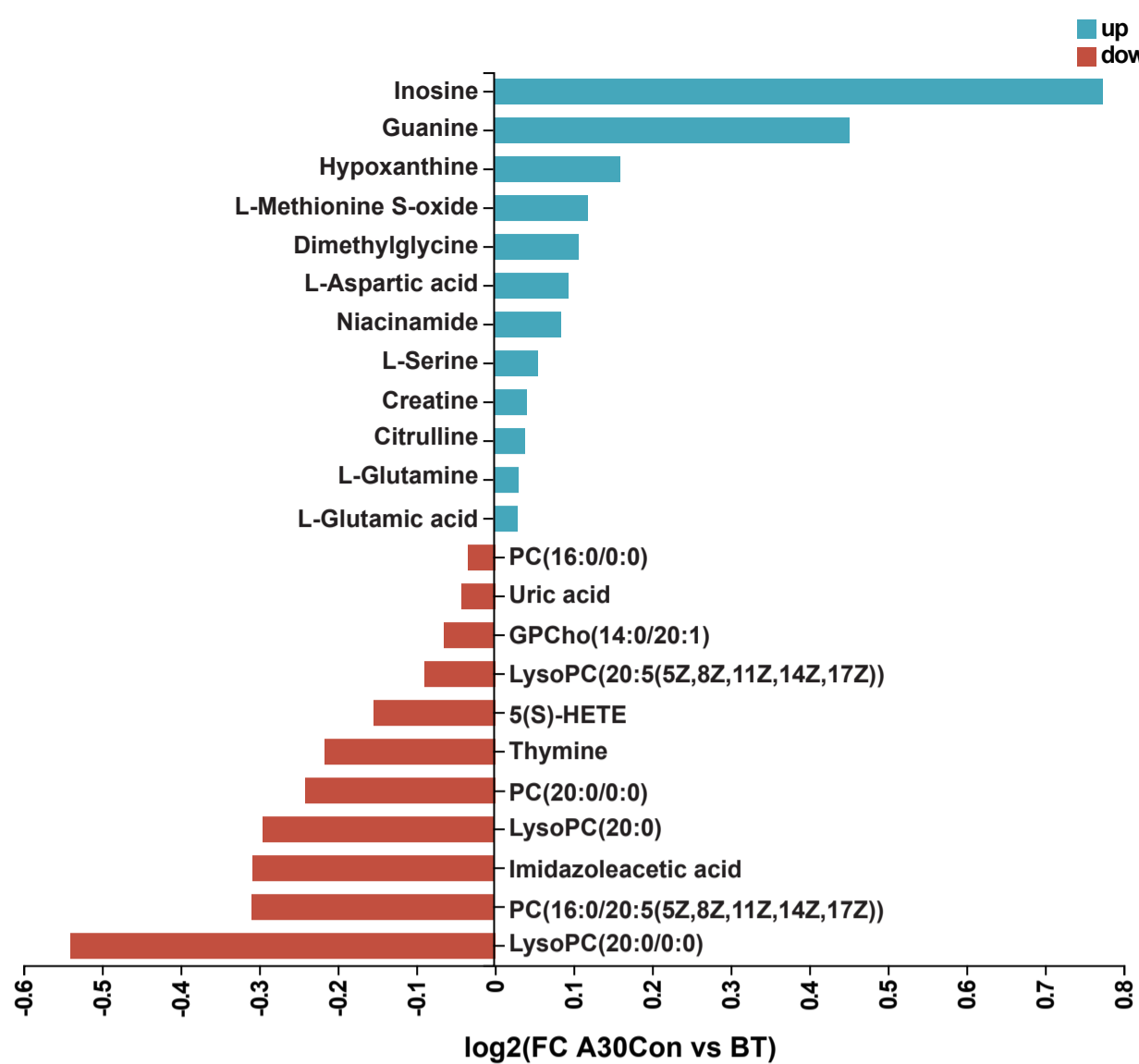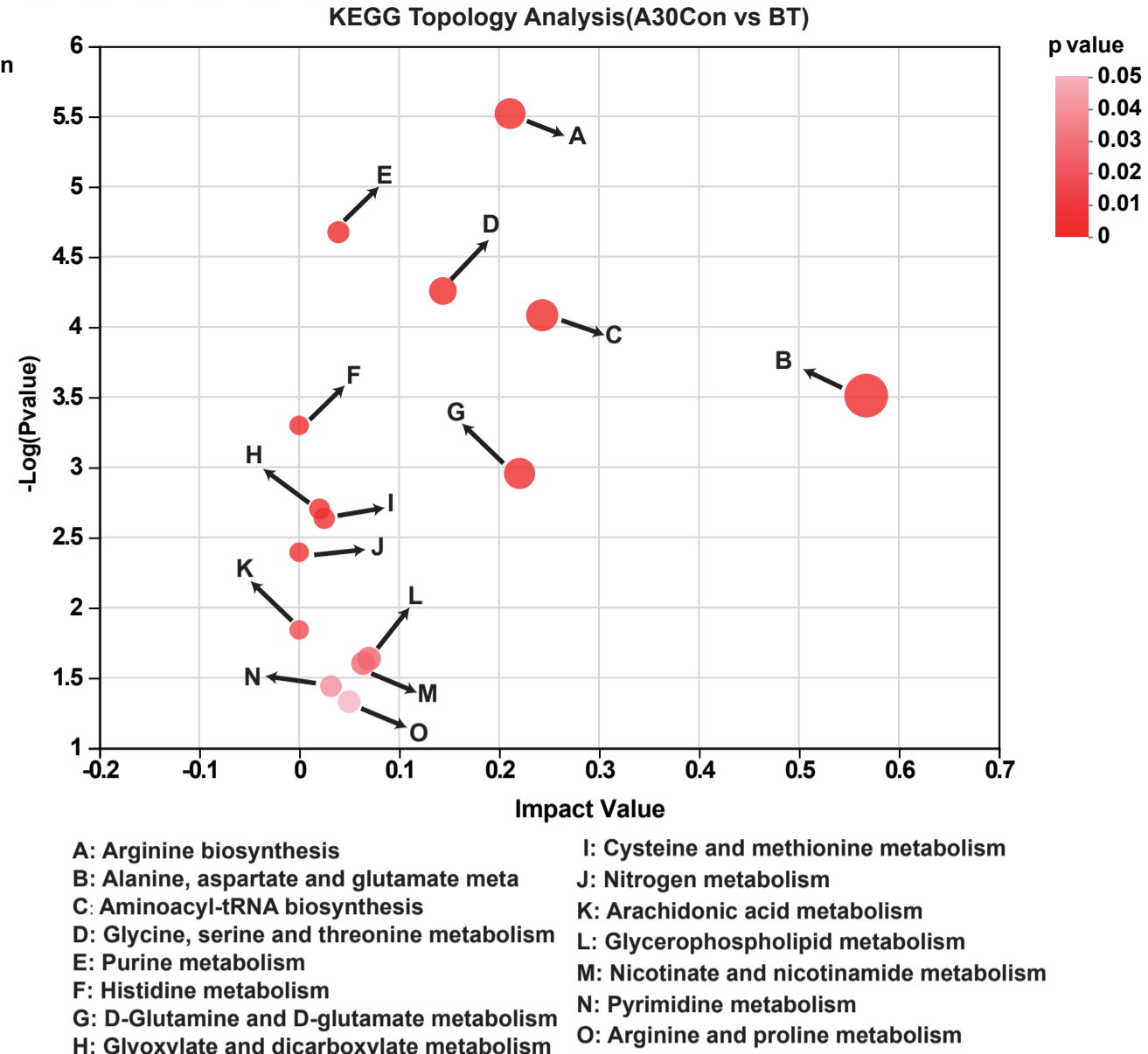

B

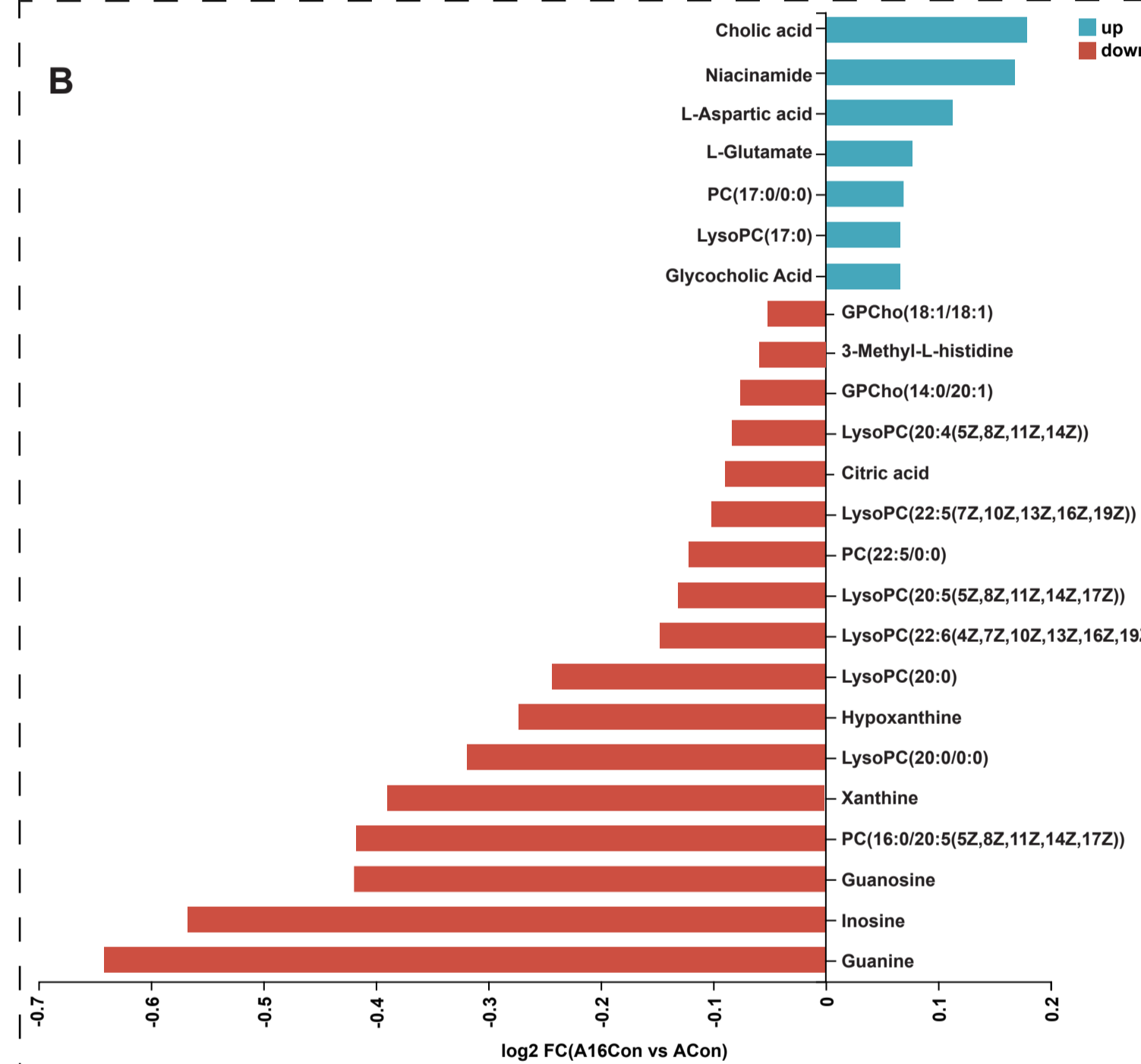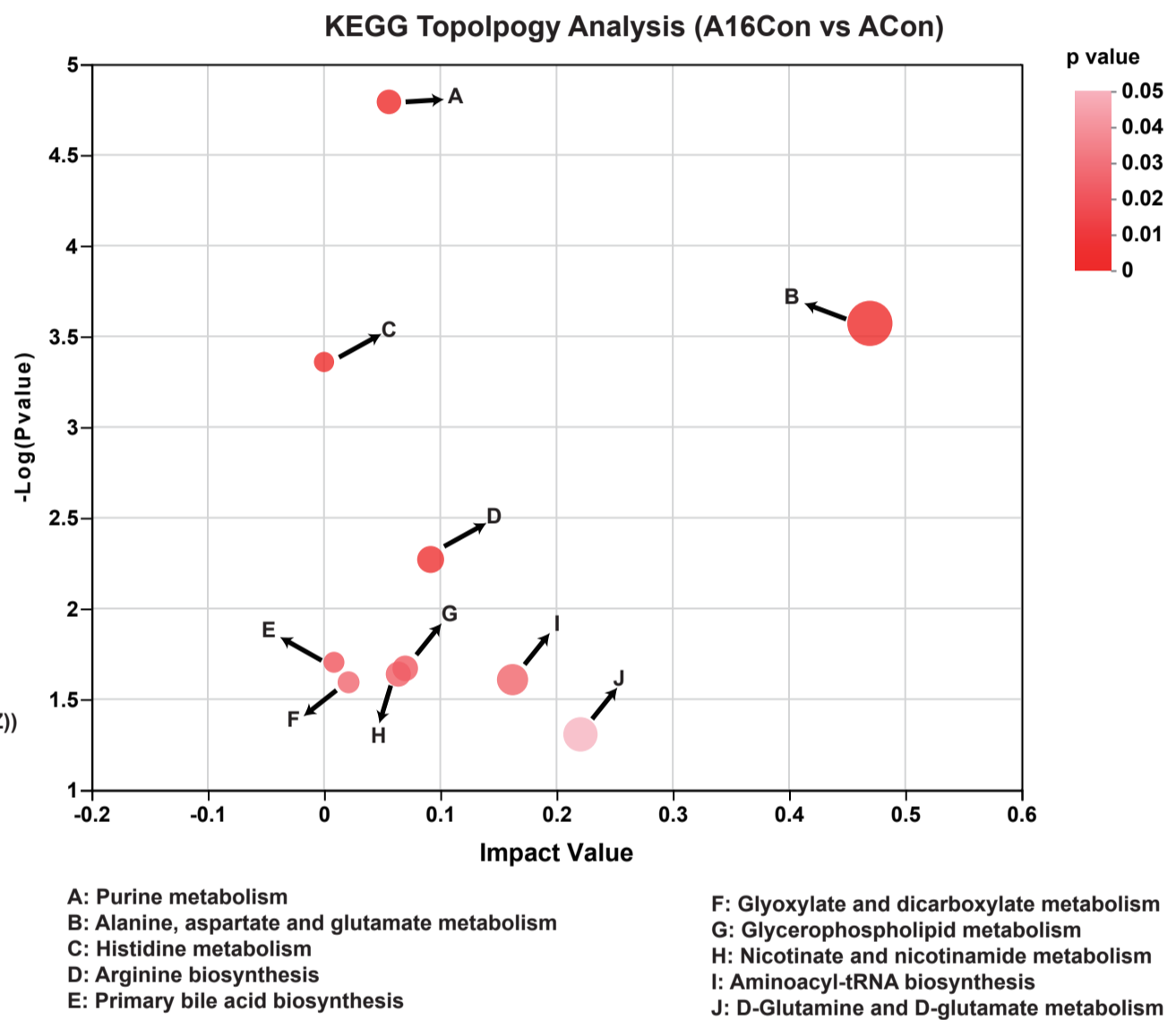

C

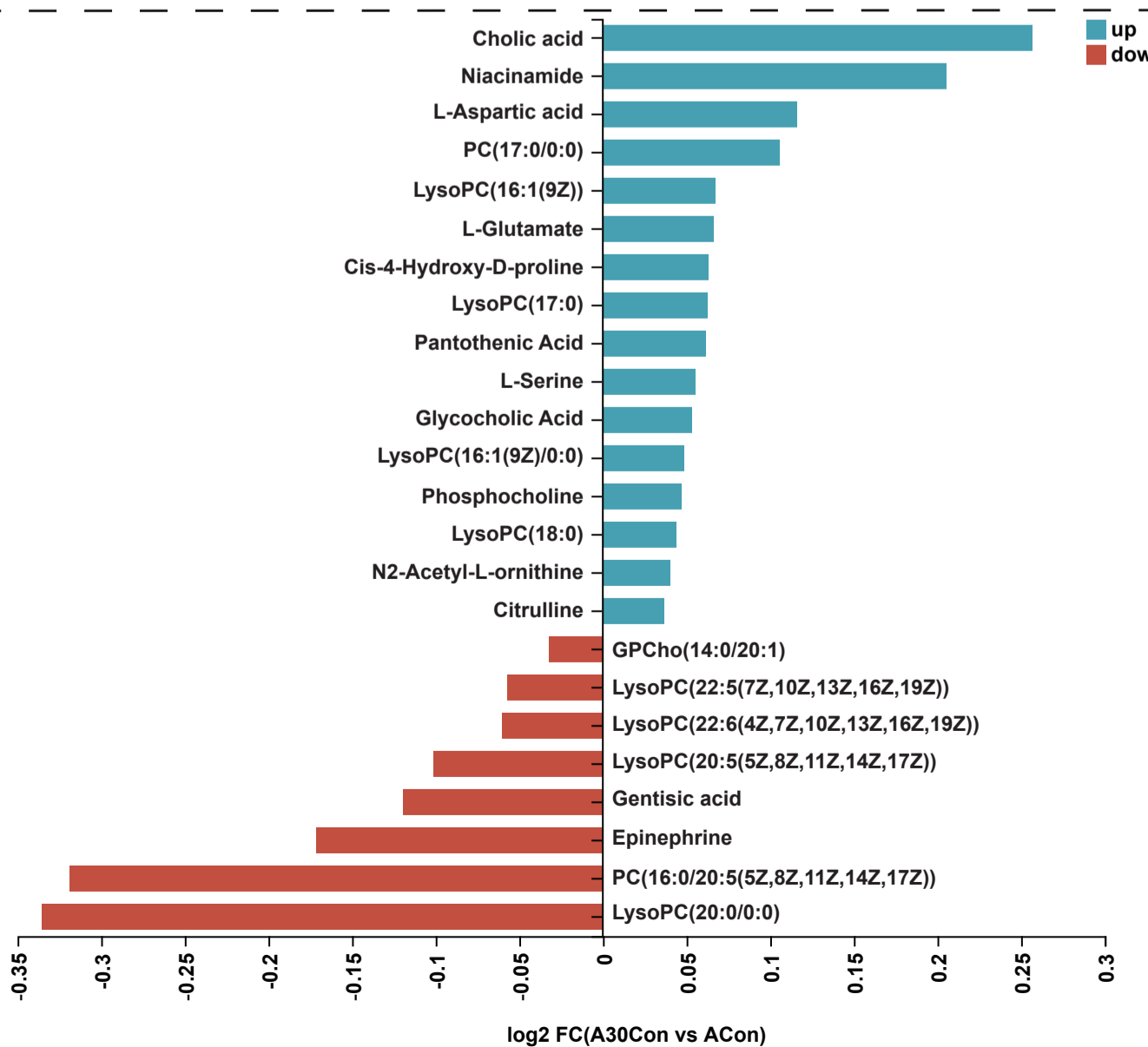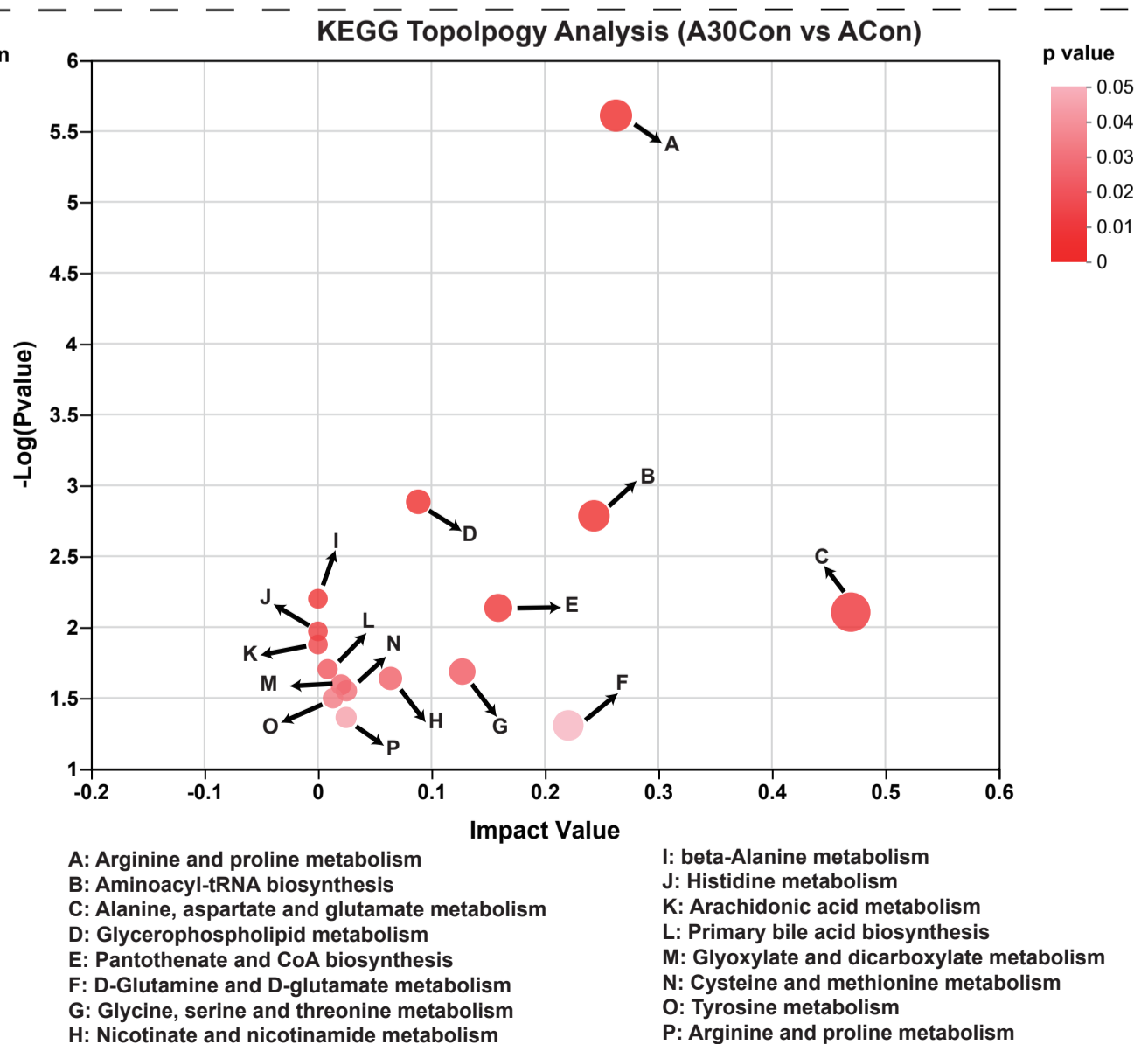

Fig. S9 Serum metabolome of A30Con vs BT, A16Con vs ACon, and A30Con vs ACon

Supplement: Supplementary file 17 — Additional file 16: Fig. S9. Serum metabolome of A30Con vs BT, A16Con vs ACon, and A30Con vs ACon. [file 40168_2023_1682_MOESM16_ESM.pdf]
