# Supplementary material for: Revealing the developmental characterization of rumen microbiome and its host in newly received cattle during receiving period contributes to formulating precise nutritional strategies
Source: Microbiome. 2023 Nov 3;11:238. doi: 10.1186/s40168-023-01682-z (PMC10623857; doi:10.1186/s40168-023-01682-z)
Supplement: Supplementary file 16 — Additional file 15: Fig. S8. Serum metabolites of BT, ACon, A16Con, and A30Con cattle categorized according to HMDB (A) and comparison based on PLS-DA (B). [file 40168_2023_1682_MOESM15_ESM.pdf]

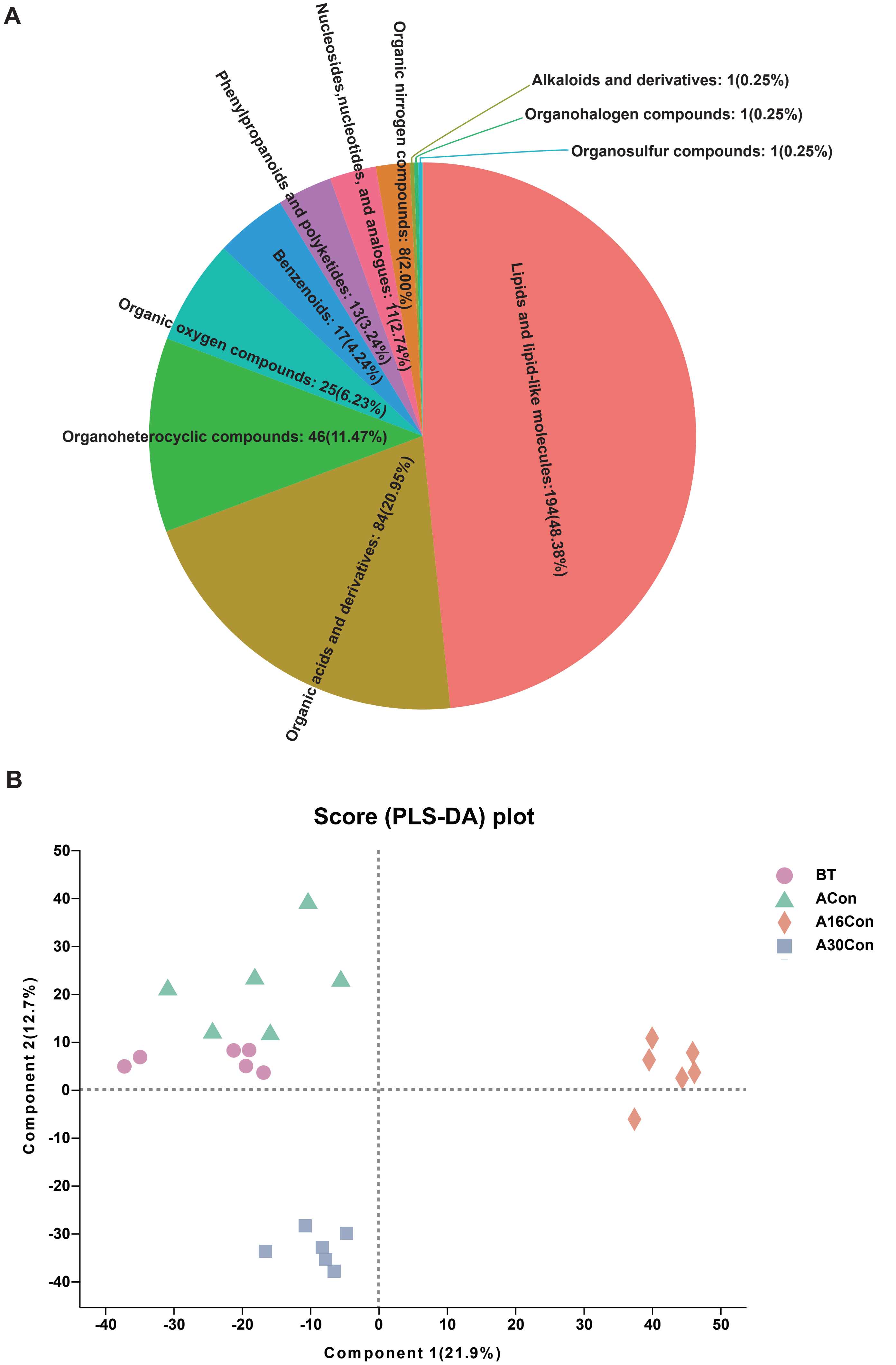

Fig. S8 Serum metabolites of BT, ACon, A16Con, and A30Con cattle categorized according to HMDB (A) and comparison based on PLS-DA (B)
