# Supplementary material for: Revealing the developmental characterization of rumen microbiome and its host in newly received cattle during receiving period contributes to formulating precise nutritional strategies
Source: Microbiome. 2023 Nov 3;11:238. doi: 10.1186/s40168-023-01682-z (PMC10623857; doi:10.1186/s40168-023-01682-z)
Supplement: Supplementary file 19 — Additional file 18: Fig. S11. Metabolic pathway difference-in-difference analysis on the pyrimidine based on metagenomics data. [file 40168_2023_1682_MOESM18_ESM.pdf]

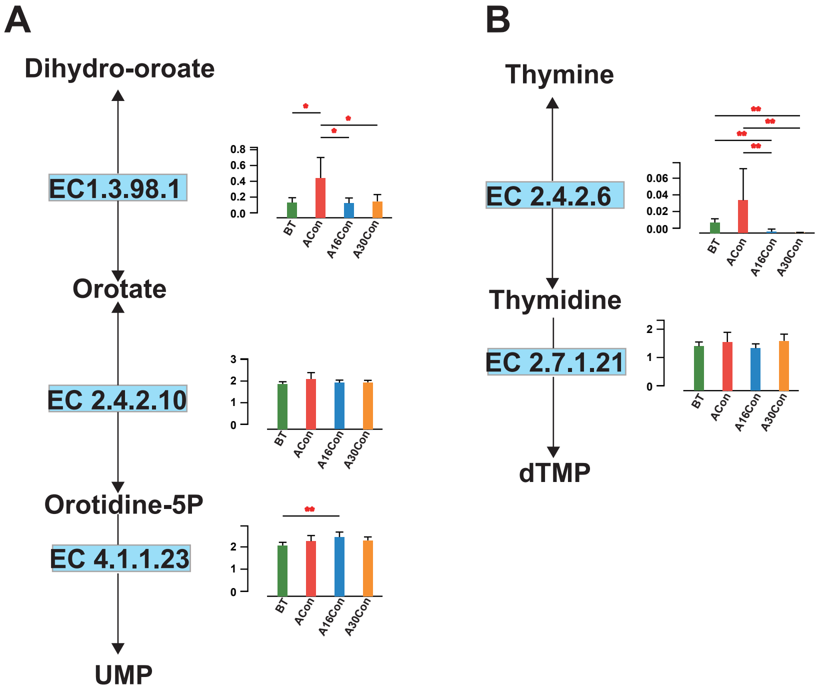

Fig. S11 Metabolic pathway difference-in-difference analysis on the pyrimidine based on metagenomics data
