# Supplementary material for: Revealing the developmental characterization of rumen microbiome and its host in newly received cattle during receiving period contributes to formulating precise nutritional strategies
Source: Microbiome. 2023 Nov 3;11:238. doi: 10.1186/s40168-023-01682-z (PMC10623857; doi:10.1186/s40168-023-01682-z)
Supplement: Supplementary file 9 — Additional file 8: Fig. S1. Profiles of rumen microbial composition of newly received cattle. [file 40168_2023_1682_MOESM8_ESM.pdf]

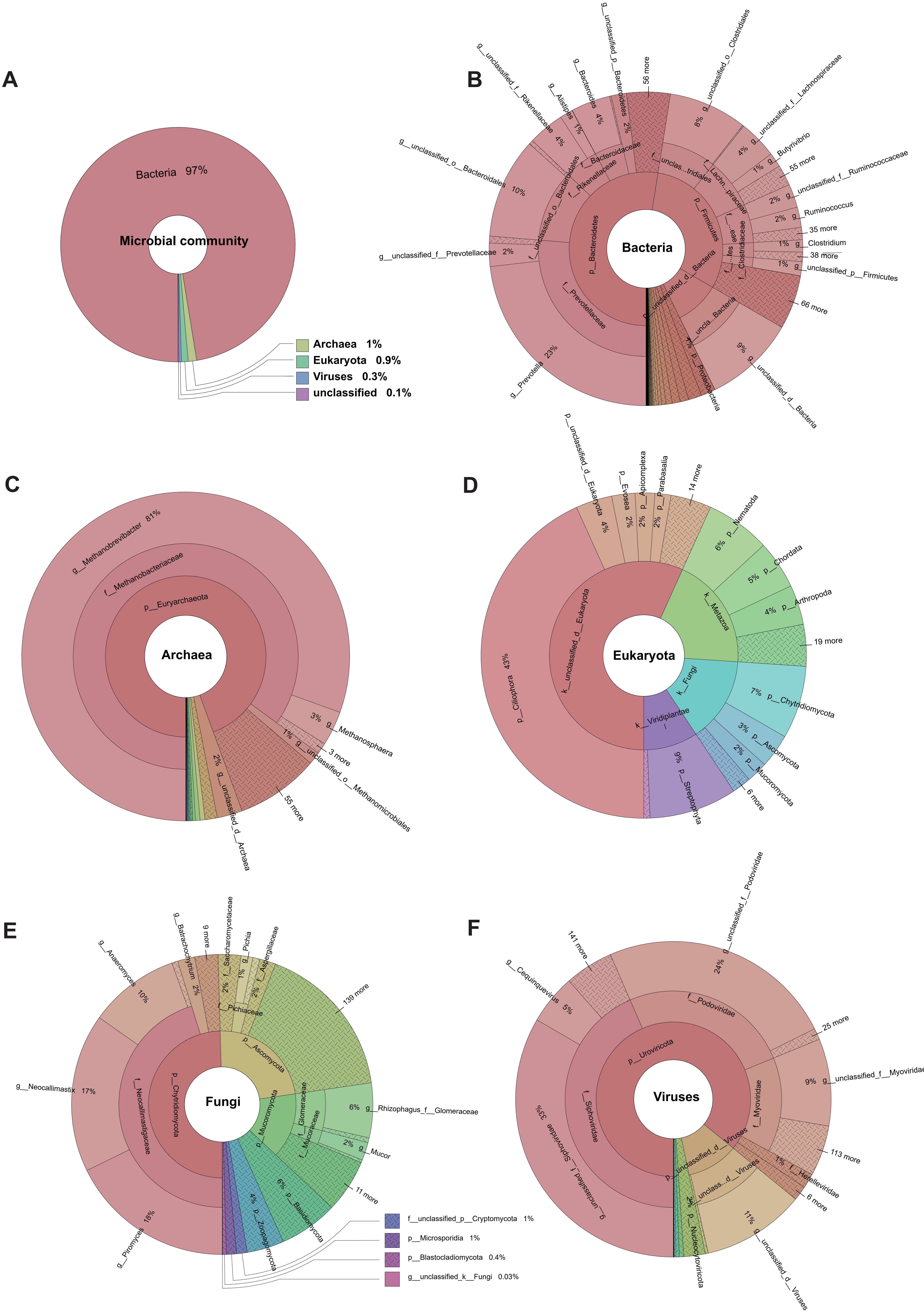

Fig. S1 Profiles of rumen microbial composition of newly received cattle
